# Baicalin ameliorates neuroinflammation-induced depressive-like behavior through inhibition of toll-like receptor 4 expression via the PI3K/AKT/FoxO1 pathway

**DOI:** 10.1186/s12974-019-1474-8

**Published:** 2019-05-08

**Authors:** Li-Ting Guo, Si-Qi Wang, Jing Su, Li-Xing Xu, Zhou-Ye Ji, Ru-Yi Zhang, Qin-Wen Zhao, Zhan-Qiang Ma, Xue-Yang Deng, Shi-Ping Ma

**Affiliations:** 1 Department of Pharmacology of Chinese Materia Medica, China Pharmaceutical University, Tongjiaxiang 24, Nanjing, 210009 Jiangsu People’s Republic of China; 2 Qinba Traditional Chinese Medicine Resources Research and Development Center, AnKang University, AnKang, 725000 People’s Republic of China

**Keywords:** Depression, Baicalin, Neuroinflammation, TLR4, FoxO1

## Abstract

**Background:**

Baicalin, which is isolated from *Radix Scutellariae*, possesses strong biological activities including an anti-inflammation property. Recent studies have shown that the anti-inflammatory effect of baicalin is linked to toll-like receptor 4 (TLR4), which participates in pathological changes of central nervous system diseases such as depression. In this study, we explored whether baicalin could produce antidepressant effects via regulation of TLR4 signaling in mice and attempted to elucidate the underlying mechanisms.

**Methods:**

A chronic unpredictable mild stress (CUMS) mice model was performed to explore whether baicalin could produce antidepressant effects via the inhibition of neuroinflammation. To clarify the role of TLR4 in the anti-neuroinflammatory efficacy of baicalin, a lipopolysaccharide (LPS) was employed in mice to specially activate TLR4 and the behavioral changes were determined. Furthermore, we used LY294002 to examine the molecular mechanisms of baicalin in regulating the expression of TLR4 in vivo and in vitro using western blot, ELISA kits, and immunostaining. In the in vitro tests, the BV2 microglia cell lines and primary microglia cultures were pretreated with baicalin and LY292002 for 1 h and then stimulated 24 h with LPS. The primary microglial cells were transfected with the forkhead transcription factor forkhead box protein O 1 (FoxO1)-specific siRNA for 5 h and then co-stimulated with baicalin and LPS to investigate whether FoxO1 participated in the effect of baicalin on TLR4 expression.

**Results:**

The administration of baicalin (especially 60 mg/kg) dramatically ameliorated CUMS-induced depressive-like symptoms; substantially decreased the levels of interleukin-1 beta (IL-1β), interleukin-6 (IL-6), and tumor necrosis factor alpha (TNF-α) in the hippocampus; and significantly decreased the expression of TLR4. The activation of TLR4 by the LPS triggered neuroinflammation and evoked depressive-like behaviors in mice, which were also alleviated by the treatment with baicalin (60 mg/kg). Furthermore, the application of baicalin significantly increased the phosphorylation of phosphatidylinositol 3-kinase (PI3K), protein kinase B (AKT), and FoxO1. The application of baicalin also promoted FoxO1 nuclear exclusion and contributed to the inhibition of the FoxO1 transactivation potential, which led to the downregulation of the expression of TLR4 in CUMS mice or LPS-treated BV2 cells and primary microglia cells. However, prophylactic treatment of LY294002 abolished the above effects of baicalin. In addition, we found that FoxO1 played a vital role in baicalin by regulating the TLR4 and TLR4-mediating neuroinflammation triggered by the LPS via knocking down the expression of FoxO1 in the primary microglia.

**Conclusion:**

Collectively, these results demonstrate that baicalin ameliorated neuroinflammation-induced depressive-like behaviors through the inhibition of TLR4 expression via the PI3K/AKT/FoxO1 pathway.

**Electronic supplementary material:**

The online version of this article (10.1186/s12974-019-1474-8) contains supplementary material, which is available to authorized users.

## Background

Depression is a life-threatening and debilitating mental disorder characterized by melancholy feelings and a lack of interest and motivation. Increasing amounts of data suggest that neuroinflammation is associated with the etiology of depression [1–3]. Preclinical and clinical research has demonstrated that central and peripheral elevation of pro-inflammation cytokines might be contributors to depressive-like behaviors [4]. Preclinical research has shown that pro-inflammation cytokines, including IL-1β, IL-6, and TNF-α, play a vital role in the onset and progression of inflammation, which subsequently induces depressive behaviors [5–7]. It has been previously shown that major depressive disorder (MDD) patients exhibited higher levels of IL-6 and TNF-α in the blood and cerebrospinal fluid (CSF) [8, 9]. In addition, the expression of IL-1β, IL-6, and TNF-α was also increased in postmortem brain samples from suicide victims that suffered from depression [10]. Most psychiatric and neurological diseases are aggravated by stress. Stress-evoked mood disorders are partially related to the dysfunction of the inflammatory system, and compelling evidence indicates that the stress-evoked elevation of inflammatory factors contributes to depression and may even deteriorate it further [3–12].

TLR4 is a pattern recognition receptor expressed in a variety of cells, including macrophages, microglia, astrocytes, and neurons [13], and has regulatory roles in the adrenal response to stress and inflammatory stimuli as well as the brain response to stress [14, 15]. The expression of TLR4 is not static because it is swiftly regulated in response to pathogens, various cytokines, and environmental stresses [16]. Activation of the TLR4 complex might underlie the pathophysiology of stress-evoked depression. This was shown by repressing stress-elevated TLR4 expression in the rodent brain and inhibiting TLR4 activity mitigated stress-induced NF-κB activation and attenuation of the upregulation of pro-inflammation cytokines both in the hippocampus and prefrontal cortex [17, 18]. Although some antidepressants exert effects by inhibiting the expression of TLR4, the exact mechanism of regulating TLR4 expression remains unclear.

**Fig. 1:**
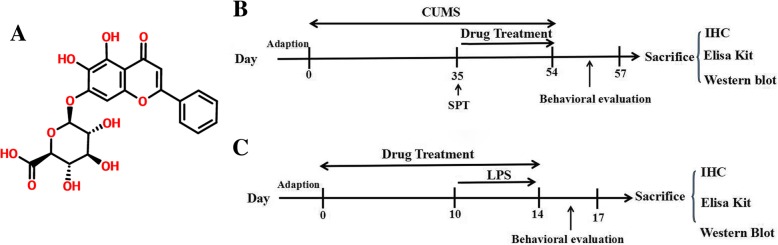
**a** The structural formula of baicalin. **b** A schematic representation of the CUMS procedure and treatments in mice. **c** A schematic representation of the LPS procedure and treatments in mice

Baicalin (β-d-glucopyranosiduronic acid, 5, 6-dihydroxy-4-oxo-2-phenyl-4H-1-benzopyran-7-yl, *M* = 446.36, Fig. 1a), which is a lipophilic flavonoid glycoside (log*P* = 1.27) [19] isolated from *Radix Scutellariae*, possesses strong biological activities including anti-inflammatory, antioxidant, and antiapoptotic effects [20]. Intriguingly, the current research has shown that baicalin exerts neuroprotective and anti-neuroinflammatory effects by regulating the activation of 5′ adenosine monophosphate-activated protein kinase (AMPK) [21] and the phosphatidylinositol 3-kinase (PI3K)/protein kinase B (AKT) pathway [10, 22]. FoxO1, which is a member of the FoxO family, has transactivation activity by binding to multiple enhancer-like elements within the TLR4 gene [23]. The transcriptional activity of FoxO1 is mediated by the PI3K/AKT pathway and other signal pathways [24]. Importantly, recent findings revealed that baicalin can be used to treat a variety of inflammatory diseases through the downregulation of TLR4 expression including LPS-induced liver inflammation [25], experimental periodontitis [26], acute pancreatitis [27], and experimental colitis [28]. Collectively, we hypothesized that baicalin might ameliorate neuroinflammation-induced depressive-like behaviors via the regulation of TLR4 expression. To investigate this hypothesis, we created chronic and acute stress tests to examine the activation of TLR4 and the levels of IL-1β, IL-6, and TNF-α in the hippocampus of depressive mice and then further investigated the possible molecular and cellular mechanisms underlying the antidepressive effects of baicalin in BV2 line cells and primary microglia cells.

## Methods

### Drugs and reagents

Baicalin (purity ≥ 98%) was obtained from Xi’an Kai Lai Biological Engineering Co., Ltd. (Xi’an, China). Fluoxetine hydrochloride (Flu) was purchased from Changzhou Siyao Pharmaceuticals Co., Ltd. (Changzhou, China). Lipopolysaccharide (LPS), 4,6-diamidino-2-phenylindole (DAPI), and compound C were bought from Sigma-Aldrich Co (St. Louis, USA). TAK-242 (a TLR4 antagonist) and LY294002 (a PI3K inhibitor) were products purchased from Apex Bio (Houston, USA). Poly-d-lysine was obtained from Sigma (MO, USA). Interleukin-1β (IL-1β), Interleukin-6 (IL-6), and tumor necrosis factor α (TNF-α) enzyme-linked immunosorbent assay (ELISA) kits were supplied by Elabscience Biotechnology Co., Ltd. (Wuhan, China). The antibodies were obtained from the cited commercial sources: anti-p-PI3K (#4228), anti-PI3K (#4292), anti-p-Akt (Ser473, #9271), anti-Akt (#9272), anti-β-actin (#4967), anti-PCNA (#13110), anti-p-AMPK (#2531), and anti-AMPK (#2603) were from Cell Signaling Technology (Beverly, MA, USA); anti-PTEN (sc-7974) and anti-p-PTEN (sc-377573) were from Santa Cruz Biotechnology (Santa Cruz, CA); anti-TLR4 (AF7017), anti-p-GSK3β (AF2016), and anti-GSK3β (AF7814) were from Affinity Biosciences (Changzhou, China); and anti-FoxO1 (ab52857), anti-p-FoxO1 (Ser 256, ab131339), and goat anti-rabbit IgG H&L (Alexa Fluor® 488, ab150077) were from Abcam (Cambridge, MA, USA).

### Animals

The experiments were performed on male ICR mice (18–22 g), which were obtained from the Experimental Animal Center in Jiangsu Province (Nanjing, China) and maintained under standard laboratory conditions. The mice were randomly housed in cages under a 12-h light/dark cycle (lights on at 7:00 a.m.), 60% humidity, and a temperature of 24 ± 1 °C with free access to water and food. All of the procedures were strictly performed in accordance with the Provision and General Recommendation of the Chinese Experimental Animals Administration Legislation and were approved by the Science and Technology Department of Jiangsu Province.

### CUMS protocol and treatments

The chronic unpredictable mild stress (CUMS) procedure was performed as described earlier with minor modifications [29–31]. After adaptation, mice in the CUMS groups were subjected to different stressors for 8 weeks as shown in Table 1. Two different stressors were applied each day, and the sequence of stressors changed every 3 days. Non-stressed mice were kept in undisturbed cages. The schematic of the experimental procedure is shown in Fig. 1b. After 5 weeks of the CUMS procedure, the sucrose preference test was performed to measure whether the CUMS model was successful.

**Table 1:** The stressors of CUMS protocol

Stressor	Duration
Water deprivation	12 h
Soiled cage	12 h
Food deprivation	12 h
Cage title (45°)	12 h
Empty bottle	12 h
Overnight illumination	12 h
Physical restrain	1 h
Cage shaking	10 min
Tail nipping	10 min
Overhang	10 min
Noise	10 min

Mice exhibiting anhedonia behavior, which is a principal symptom of the CUMS model [31, 32], were randomly assigned to four groups (*n* = 15 in each group): CUMS group, CUMS+Flu (fluoxetine, 20 mg/kg [12], i.g.) group, CUMS+BA30 (baicalin, 30 mg/kg, i.g.) group, and CUMS+BA60 (baicalin, 60 mg/kg, i.g.) group. Non-stressed mice were randomly divided into two groups (*n* = 15 in each group): the control group and the control+BA60 (baicalin, 60 mg/kg, i.g.). The doses of baicalin were selected based on earlier reports [33, 34]. From the sixth week, baicalin or Flu was intragastrically administered once daily for the subsequent 3 weeks and chronic treatments and CUMS stimulus were continued throughout the treatment period. Mice in the control group and the CUMS group were given an equal volume of double distilled water.

In addition, another CUMS protocol was designed to explore the further mechanism of baicalin. The mice were administered the aforementioned CUMS procedure and assigned to four groups (*n* = 15 in each group): control group (non-stress), CUMS group, CUMS+BA60 (baicalin, 60 mg/kg, i.g.), and CUMS+LY+BA60 (LY294002, 6 μg, intranasally; baicalin, 60 mg/kg, i.g.). LY294002 was administered intranasally to mice 1 h before the administration of baicalin. The administration method and dose of LY294002 were taken from previous reports [35, 36]. After a 3-week treatment, the behavioral tests were performed and the animals were sacrificed after behavioral experiments.

### LPS protocol and treatments

After the adaptation, male mice were randomly allocated into four groups (*n* = 12 in each group): the control group, LPS group, LPS+TAK (TAK-242, 3 mg/kg, i.p.) group, and LPS+BA60 (baicalin, 60 mg/kg, i.g.). TAK-242 or baicalin was administered once daily for 14 consecutive days, and LPS was administered intraperitoneally once a day for the subsequent 5 days from the tenth day as shown in Fig. 1c. The doses of TAK-242 were chosen based on earlier reports [37, 38]. LPS was injected (i.p.) at the dose of 0.83 mg/kg and according to the previous studies [39–41]. The behavioral tests were performed 12 h after the last LPS administration.

### Behavioral evaluation

All of the behavioral tests were conducted in parallel in all of the animals. The protocol followed the sequence of sucrose preference test (SPT), open field test (OFT), tail suspension test (TST), and forced swimming test (FST) as previously described [8–10]. This sequence was chosen to expose the animals to the same experimental conditions; however, mice were taken in a random sequence to avoid testing an entire group at once. All of the behavioral tests were conducted blindly with respect to the surgery and drug administration and under conditions of dim light and low noise.

### Sucrose preference test

Anhedonia (as a core symptom of CUMS mice) was monitored using the SPT as previously described [42, 44]. Briefly, the mice were acclimatized to a 1% sucrose solution (*w*/*v*) for 24 h before the test. After adaptation, the mice were deprived of water and food for 12 h. Then, the mice were housed individually and given two bottles with tap water and a 1% sucrose solution for 12 h, and the positions of the two bottles were mutually exchanged after 6 h to avoid side preference. The sucrose preference (SP) value was calculated as: SP (%) = sucrose intake (g)/[sucrose intake (g) + water intake (g)] × 100%.

### Open field test

The OFT was generally used to evaluate the locomotor activity, and the test was performed as previously described [34]. Mice were individually placed into the center of an open field apparatus and permitted free exploration for 6 min. After two minutes of adaptation to the apparatus, the number of crossings was recorded during the next 4 min. The device was cleaned with ethanol after each test.

### Tail suspension test

The TST was conducted based on earlier methodology [43]. Each mouse was suspended upside down for 6 min. After 2 min of adaptation, the immobility durations over the next 4 min were measured.

### Forced swimming test

The FST was conducted as the previously described methodology [45, 46]. The apparatus was a hyaline cylinder (15 cm diameter) filled with 22–25 °C water to a depth of 20 cm. The mice were exposed to a training period of 15 min, and 24 h later, the mice were individually placed in the water to swim for 6 min and the immobile time was calculated during the final 4 min.

### Immunohistochemistry and Nissl staining

The mice were deeply anesthetized with 10% (*w*/*v*) chloral hydrate and transcardially perfused with 100 mL of phosphate buffer saline (PBS, pH 7.3) followed by 50 mL of 4% (*w*/*v*) paraformaldehyde in PBS [47]. The brains were removed and immersed in 10% formalin overnight at 4 °C and then processed for paraffin embedding and sectioned into coronal slices of 6 μm [30]. The procedure was performed as previously described [48]. Briefly, the paraffin sections were deparaffinized, rehydrated, and blocked. Subsequently, the sections were incubated with rabbit anti-TLR4 (1:100) overnight for 4 °C. The sections were washed with PBS and incubated with goat anti-rabbit secondary antibody at room temperature for 1 h. The samples were rinsed, and a 3, 3-diaminobenzidine color reaction was performed. After dehydration, the sections were transparentized. The images of the hippocampal CA1 region were collected using light microscopy and quantified by Image-Pro Plus 6 (IPP 6).

For Nissl staining [49], the brain slices were dewaxed with xylene and hydrated in a graded series of alcohol. Then, the samples were stained by a Nissl solution for 10 min, washed with distilled water, dehydrated by gradient alcohol, transparentized by xylene, and mounted by neutral balsam. Next, the sections were observed and photographed under a microscope. The numbers of positive cells were quantified in the CA1 hippocampal subfield with IPP 6.

### Cell culture and treatments

The murine BV2 microglial cells were cultured in Dulbecco’s modified Eagle’s medium (DMEM, Gibco) supplemented with 10% fetal bovine serum (FBS, Biological Industries) and incubated at 37 °C in a humidified incubator under 5% CO_2_. The cells were pretreated with baicalin (100 μM) and LY292002 (10 μM) for 1 h followed by post-incubation with LPS (1 μg/mL) for 24 h. The concentration of baicalin was selected by the MTT assay, and the concentration of LPS and LY294402 was considered according to previously described studies [50, 51].

### Primary mouse microglial cell culture

Primary microglia cultures were prepared following standard methods [52, 53] (details in Additional file 1) and cultured in the Dulbecco’s modified Eagle’s medium nutrient mixture F-12 (DMEM/F12, Gibco) containing 10% FBS and antibiotics (40 U/mL penicillin and 40 μg/mL streptomycin). After isolation from mixed glial cultures, the microglia cells were allowed to rest overnight prior to the treatments.

### Transfection

For small interfering RNA (siRNA) transfection, primary microglia cells were transfected with FoxO1-specific siRNA (GenePharma, Shanghai, China) or negative control siRNA for 5 h via Lipofectamine 2000 (Invitrogen, Carlsbad, CA, USA) with Opti-MEM Reduced-Serum Medium (Gibco). Then, the medium was replaced with regular culture medium containing serum and the cells were cultured for 48 h. Next, the microglia cells were exposed to LPS (1 μg/mL) with or without baicalin (l00 μM). A western blot analysis and immunofluorescence assay were used to evaluate the efficiency of siRNA.

### Immunocytochemistry

BV2 cells and primary microglia cells were washed with cold PBS and fixed with 4% paraformaldehyde in PBS for 20 min and then permeabilized with 0.2% Triton X-100 for 15 min. Non-specific binding was blocked by incubating the cells in PBS containing 10% BSA. Cells were incubated with rabbit-anti-FoxO1 (1:100) or anti-TLR4 (1:100) overnight for 4 °C. After three washings, the cells were incubated with goat anti-rabbit IgG H&L (Alexa Fluor® 488, 1: 200) at 37 °C for 2 h and the nuclei were stained with DAPI for 10 min. Fluorescent images were obtained using a confocal microscope.

### Enzyme-linked immunosorbent assay

The levels of IL-1β, IL-6, and TNF-α in the hippocampus of CUMS mice and the cell culture medium were measured with ELISA kits following the manufacturer’s instructions. Data are shown in picograms per milligram protein (pg/mg prot).

### Cytoplasmic and nuclear protein extraction

The cytoplasmic and nuclear proteins were extracted from fresh hippocampal tissues of CUMS mice and LPS-treated BV2 cells using nuclear and cytoplasmic protein extraction kits (product no: P0028, Beyotime Institute of Biotechnology, Shanghai, China). The extraction processes were strictly performed according to the manufacturer’s instructions. The supernatants of the cytoplasmic and nuclear protein extractions were collected for subsequent experiments.

### Western blot analysis

The proteins were extracted from the hippocampal tissues of CUMS mice, LPS-treated mice, and the treated cells, and the concentrations of the supernatants were quantified by the bicinchoninic acid assay according to the manufacturer’s instructions (Beyotime, Nantong, China). The samples were separated by 10% SDS-PAGE and transferred onto polyvinylidene difluoride (PVDF) membranes and then blocked with 5% skim milk in TBS-T (TBS containing 0.1 ‰ Tween-20). The membranes were incubated with the following primary antibodies: anti-TLR4 (1:1000), anti-p-PTEN (1:800), anti-PTEN (1:800), anti-p-PI3K (1:1000), anti-PI3K (1:1000), anti-p-AKT (1:1000), anti-AKT (1:1000), anti-p-FoxO1 (1:800), anti-FoxO1 (1:1000), anti-p-AMPK (1:1000), anti-AMPK (1:1000), anti-GSK3β (1:1000), anti-p-GSK3β (1:800), anti-β-actin (1:1000), and anti-PCNA (1:1000) at 4 °C overnight. After incubation with anti-rabbit or anti-mouse secondary antibodies for 90 min, the membranes were scanned by an imaging system (Bio-Rad, Hercules, CA, USA) and then quantified using ImageJ software (National Institutes of Health, USA).

### Statistical analysis

Data are represented as the mean ± SEM. The statistical analysis was conducted using the one-way ANOVA test followed by Tukey’s test. A *p* value < 0.05 was considered significant. All of the data figures were obtained by GraphPad Prism 5.01 statistical software.

## Results

### Baicalin reversed stress-induced depressive-like behavior by alleviating neuroinflammation

**Fig. 2:**
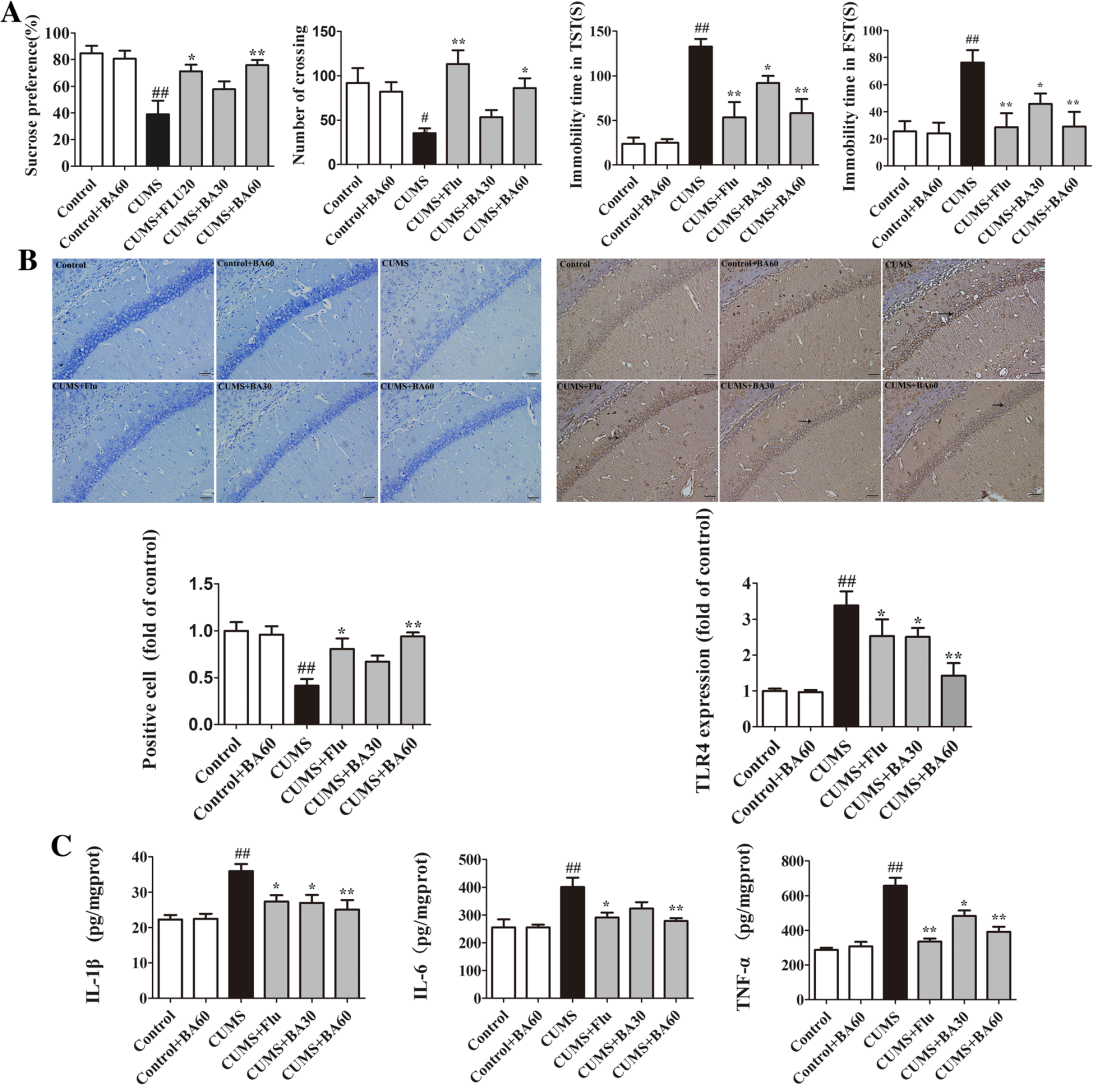
Baicalin reversed CUMS-induced depressive behavior by alleviating neuroinflammation. **a** The effects of baicalin treatment on the behavioral evaluation in CUMS-induced mice (*n* = 12). The sucrose preference test (**a**, first), the crossing number in OPT (**a**, second), the immobility time in TST (**a**, third), and the immobility time in FST (**a**, fourth). **b** The pathological section results of baicalin’s effects on CUMS mice (*n* = 3). Nissl staining in the hippocampal CA1 region and its statistical analysis (**b**, first), and immunohistochemistry staining of TLR4 in area CA1 of the hippocampus and its statistical analysis (**b**, second) (scale bar 20 μm, *n* = 3 in each test). **c** The successive effects of baicalin on IL-1β, IL-6, and TNF-α in the hippocampus of CUMS mice (*n* = 8). All of the data are presented as means ± SEM. ^#^*p* < 0.05, ^##^*p* < 0.01 vs. control; *p < 0.05, **p < 0.01 vs. CUMS

To investigate the effects of baicalin on CUMS-induced depressive-like behaviors of mice, we performed behavioral tests including SPT, OFT, TST, and FST as successively shown in Fig. 2a. In SPT and compared with the control mice, the consumption of sucrose was significantly reduced in CUMS-exposed mice (*p* < 0.01, *F*[5,66] = 6.887). However, treatment with baicalin (60 mg/kg, *p* < 0.01) and Flu (*p* < 0.05) greatly reversed the abatement of the percentage of the sucrose preference, which reflected anhedonia (Fig. 2a, the first). Then, the locomotor activities were examined in the OFT. The CUMS mice showed a significant reduction in the number of crossings compared with the control group (*p* < 0.01, *F*[5,66] = 6.122). Treatment with baicalin (60 mg/kg, *p* < 0.05) and Flu (*p* < 0.01) markedly improved the CUMS-induced lessening of locomotor activity (Fig. 2a, the second). No significant differences between the control group and the control+BA60 group indicated that baicalin had no significant effect on central excitability in normal mice. The desperate behaviors of mice were measured by recording the immobility time in the TST and FST. The CUMS mice observably increased the duration of immobility in TST (*p* < 0.01, *F*[5,66] = 14.010, Fig. 2a, the third) and FST (*p* < 0.01, *F*[5,66] = 4.734, Fig. 2a, the fourth) compared to the control mice. As expected, the administration of baicalin (30 mg/kg, *p* < 0.05; 60 mg/kg, *p* < 0.01) and Flu (*p* < 0.01) remarkably reduced the immobility time compared with the CUMS group. These results indicate that baicalin ameliorates CUMS-induced depressive-like behaviors.

Nissl staining was used to observe the morphological changes of the hippocampal neural cells in response to baicalin against CUMS-evoked neurotoxicity in mice. As shown in Fig. 2b first, neural cells in the hippocampal CA1 region were arrayed regularly and closely, and the Nissl bodies were clear in the control group. However, an observable ratio of neurons in the CUMS group was damaged with irregular and loose distribution as well as cytoplasm pyknosis and Nissl body disintegration. The numbers of Nissl-positive cells were observably reduced in the hippocampal CA1 region in the model mice compared with the control group (*p* < 0.01, *F*[5,12] = 7.397). In contrast, the severity of neuronal damage and the decrease of Nissl-positive cell numbers induced by CUMS were markedly ameliorated by baicalin (60 mg/kg, *p* < 0.01) and Flu (*p* < 0.05).

The above results show that baicalin pretreatment alleviated the CUMS-induced injury of the hippocampal neurons. We investigated the effects of baicalin on pro-inflammatory cytokines production in the hippocampus of CUMS mice. As shown in Fig. 2c, the levels of IL-1β (*p* < 0.01, *F*[5,42] = 6.410), IL-6 (*p* < 0.01, *F*[5,42] = 6.156), and TNF-α (*p* < 0.01, *F*[5,42] = 23.31) were evidently elevated in the CUMS group versus the control group, whereas these effects were significantly reversed by the treatment with Flu (*p* < 0.05) and baicalin (60 mg/kg, *p* < 0.01). These data reveal that baicalin repressed the neuroinflammation in CUMS mice by downregulating hippocampal pro-inflammatory cytokines.

As described in Fig. 2b second, immunohistochemistry results showed that CUMS exposure also increased the expression of TLR4 in the hippocampal CA1 region versus the control group (*p* < 0.01, *F*[5,12] = 10.15). Baicalin (30 mg/kg, *p* < 0.05; 60 mg/kg, *p* < 0.01) and Flu (*p* < 0.05) administration observably inhibited the TLR4 expression compared with CUMS mice. Surprisingly, these results were mainly consistent with the anti-neuroinflammatory effect of baicalin, which indicates that TLR4 might play a role in this result.

### Role of TLR4 in the effect of baicalin in alleviating LPS-induced neuroinflammation

To clarify the role of TLR4 in the anti-neuroinflammatory effect of baicalin on mice, we employed LPS (a main TLR4 ligand) to trigger inflammatory responses. TAK-242 is a specific inhibitor of TLR4 that works through disrupting the interactions of TLR4 with its adaptor molecules [13, 52]. We used TAK-242 as a positive pharmacological tool to block the activity of TLR4.

**Fig. 3:**
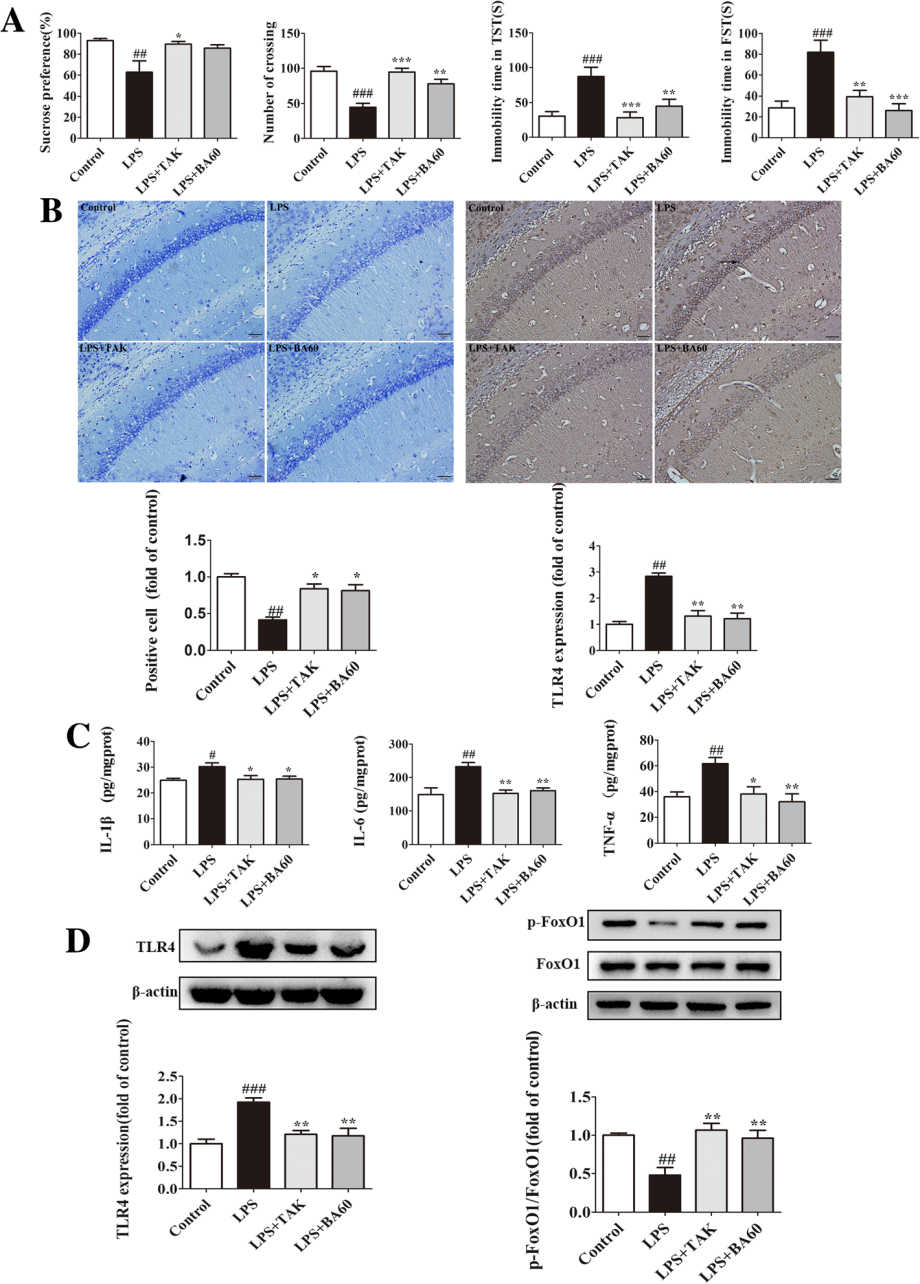
The role of TLR4 in alleviating neuroinflammation of baicalin. Prophylactic treatment of TAK-242 or baicalin inhibited LPS-induced neuroinflammation as well as depressive-like behaviors. **a** Pretreatment of TAK-242 or baicalin ameliorated LPS-induced depressive behaviors, which were successively measured by SPT, OPT, TST, and SFT (*n* = 12 in each test). **b** Representative pathological images of Nissl staining and immunohistochemistry staining of TLR4 in the hippocampal CA1 subregion (*n* = 3 in each test; scale bar 20 μm). **c** The levels of IL-1β, IL-6, and TNF-α in the hippocampus were measured by ELISA kits (*n* = 8). **d** The levels of TLR4 and p-FoxO1 in the hippocampus were measured by western blotting and were quantified and normalized with their respective β-actin or total FoxO1 levels (*n* = 4). Data were normalized to the control and presented as means ± SEM. ^#^*p* < 0.05, ^##^*p* < 0.01, ^###^*p* < 0.005 vs. control; *p < 0.05, **p < 0.01, ***p < 0.005 vs. LPS. TAK, TAK-242

As shown in Fig. 3a, the data of behavioral evaluations explicated that the activation of TLR4 by LPS evoked and exacerbated the depressive-like behaviors of mice. Compared to the control, LPS-injected mice showed a lower percentage of sucrose preference in SPT (*p* < 0.01, *F*[3,44] = 4.000), a lower locomotor activity in OPT (*p* < 0.005, *F*[3,44] = 16.100), and a longer duration of immobility in TST and FST (*p* < 0.005, *F*[3,44] = 7.628; *p* < 0.005, *F*[3,44] = 10.540, respectively). However, in comparison with the LPS group, the sucrose preference levels were higher in both LPS+TAK (*p* < 0.05) and the LPS+BA60 group. Similarly, mice in the LPS+TAK and LPS+BA60 groups were more active in OPT (*p* < 0.005; *p* < 0.01, respectively) versus mice in the LPS group. Moreover, shorter immobility times in TST were observed in the LPS+TAK (*p* < 0.005) and LPS+BA60 (*p* < 0.01) groups and shorter immobility durations in FST were expressed in the LPS+TAK (*p* < 0.01) and LPS+BA60 (*p* < 0.005) groups when compared to the LPS group.

As presented in Fig. 3c, the activation of TLR4 by LPS observably promoted the production of pro-inflammatory cytokines in the hippocampus of LPS-treated mice. In parallel with the results of behavioral evaluations, the LPS injection greatly enhanced the levels of IL-1β (*p* < 0.05, *F*[3,28] = 4.292), IL-6 (*p* < 0.01, *F*[3,28] = 8.498), and TNF-α (*p* < 0.01, *F*[3,28] = 6.870) in the hippocampus of the LPS group of mice versus the control group. The TAK-242 lessened the expression of IL-1β, IL-6, and TNF-α by blocking the biological effect of TLR4. In addition, the baicalin treatment represented the same efficacy with TAK-242 administration in LPS-induced neuroinflammation.

Data from Nissl staining are shown in Fig. 3b first. The Nissl bodies became small and indistinct, and the number of Nissl-positive cells significantly lessened in the hippocampal CA1 region of LPS-treated mice (*p* < 0.01, *F*[3,8] = 17.490) versus the control group. In addition, the treatment with TAK-242 (*p* < 0.05) and baicalin (*p* < 0.05) augmented the number of Nissl-positive cells, which indicated that TAK-242 and baicalin reduced the LPS-induced injury of hippocampal neurons.

Immunohistochemistry staining of TLR4 in area CA1 of the hippocampus showed that LPS stimulation elicited the activation of TLR4 and upregulated its expression as exhibited in Fig. 3b second (*p* < 0.01, *F*[3,8] = 24.65). TAK-242 inhibited LPS-induced TLR4 expression by blocking the TLR4 signaling pathway (*p* < 0.01). Similar to TAK-242, baicalin also downregulated the expression of TLR4 induced by LPS (*p* < 0.01).

Previous research showed that FoxO1 binds to multiple enhancer-like elements within the TLR4 gene and regulates the transcription of TLR4 [23]. We further investigated the interaction between TLR4 and FoxO1 in LPS-treated mice. As shown in Fig. 3d, LPS injection significantly enhanced the expression of TLR4 (*p* < 0.005, *F*[3,12] = 12.28) and the activity of FoxO1 by downregulating its phosphorylation (*p* < 0.01) in the hippocampus. TAK-242 blocked the LPS-induced elevation of TLR4 expression (*p* < 0.01) and the activity of FoxO1 through upregulation of the level of p-FoxO1 (*p* < 0.01). Furthermore, baicalin also decreased the expression of TLR4 (*p* < 0.01) and the activity of FoxO1. These data indicate that the blocking of TLR4 was accompanied by a higher level of p-FoxO1 and a lower transcriptional activity of FoxO1.

The activation of TLR4 could trigger neuroinflammation and evoke depression-like behaviors. Inhibition of the TLR4 signaling pathway by TAK-242 alleviated neuroinflammation and depressive behaviors induced by LPS. Baicalin ameliorated LPS-induced neuroinflammation and reversed LPS-evoked depressive-like behaviors by downregulating the expression of TLR4. Furthermore, FoxO1 might be involved in the effect of baicalin on TLR4 expression. This evidence indicates that TLR4 has a vital role in the alleviation of neuroinflammation and depressive-like behaviors by baicalin.

### Baicalin reduced CUMS-induced TLR4 expression and inflammatory responses by activating the PI3K/AKT pathway in mice

PI3K/AKT is the primary signaling transduction pathway responsible for the synthesis and production of pro-inflammatory mediators and can even regulate the expression of TLR4 [54–56]. We treated LY294002 (a PI3K/Akt pathway inhibitor) 1 h before the administration of baicalin in CUMS mice to investigate whether baicalin downregulated the expression of TLR4 in CUMS mice via the PI3K/AKT pathway.

**Fig. 4:**
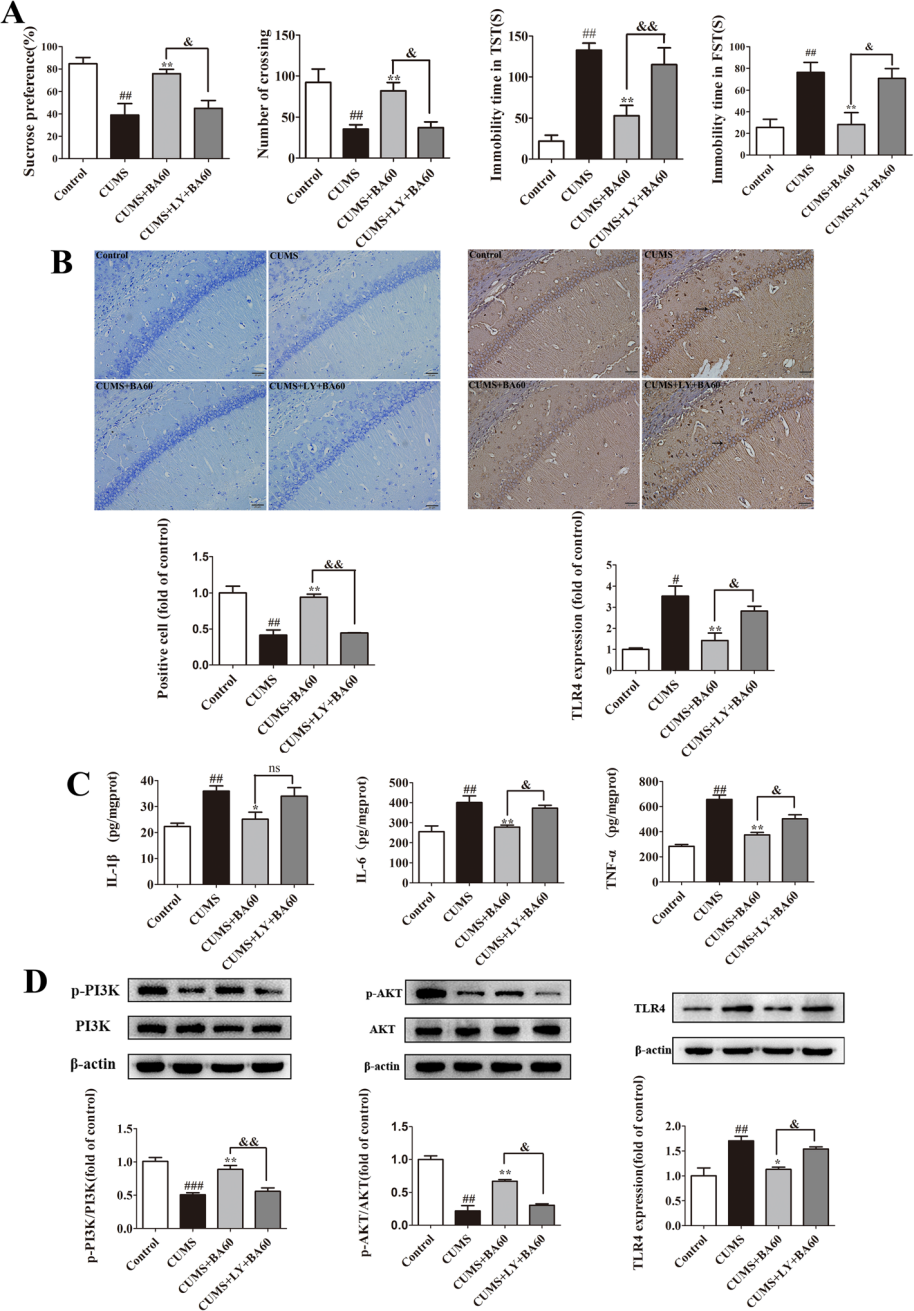
Baicalin reduced the CUMS-induced TLR4 expression and inflammatory response by activating PI3K/AKT. LY294002 was employed to abolish the effect of baicalin on PI3K/AKT in CUMS mice, and the mice were allocated into four groups: the control, CUMS, CUMS+BA60 and CUMS+LY+BA60. **a** The behavioral evaluation results including SPT, OPT, TST, and OPT (*n* = 12). **b** The Nissl staining results in the hippocampal CA1 region and the statistical analysis (**b**, first) and TLR4 immunohistochemistry staining in the hippocampal CA1 subregion and its statistical analysis (**b**, second) (scale bar 20 μm; *n* = 3 in each experiment). **c** The levels of IL-1β, IL-6, and TNF-α in the hippocampus were determined by ELISA kits (*n* = 8). **d** The western blotting results of p-PI3K, p-AKT, and TLR4 were quantified (*n* = 4). Data were normalized to the control and presented as means ± SEM. ^#^*p* < 0.05, ^##^*p* < 0.01, ^###^*p* < 0.005 vs. control; *p < 0.05, **p < 0.01 vs. CUMS; ^&^*p* < 0.05, ^&&^*p* < 0.01 vs. CUMS+BA60. LY, LY294002; BA60, baicalin treatment with 60 mg/kg

The efficacy of baicalin treatment on CUMS mice was dramatically reversed by the prophylactic administration of LY294002, which is explicated in the behavioral tests in Fig. 4a. The baicalin administration greatly attenuated depressive-like behaviors observed in CUMS-induced mice. However, in comparison with the CUMS+BA60 group, the CUMS+LY+BA60 group mice showed a lower percentages of sucrose preference in SPT (*p* < 0.05, *F*[3,44] = 9.594), a lower locomotor activity in OPT (*p* < 0.05, *F*[3, 44] = 8.022), and a longer duration of immobility in TST (*p* < 0.01, *F*[3,44] = 18.00) and FST (*p* < 0.05, *F*[3,44] = 9.860). These data indicate that the antidepression effect of baicalin in CUMS mice was blocked by LY294002.

In addition, baicalin inhibited the CUMS-induced production of IL-1β, IL-6, and TNF-α in hippocampal tissues, but LY294002 (*p* < 0.05 in IL-6 and TNF-α) abolished the anti-neuroinflammatory effects of baicalin (Fig. 4c).

As the Nissl staining results showed in Fig. 4b first, the change of the neuronal morphology and the reduction of Nissl-positive cells in the hippocampal CA1 subregion of CUMS mice were ameliorated by the baicalin treatment (*p* < 0.01, *F*[3,8] = 24.53). The protective effect of baicalin could be abrogated by the pretreatment of LY294002 (*p* < 0.01). Meanwhile, the increase of TLR4 induced by CUMS exposure in the CA1 region of the hippocampus was remarkably lessened by baicalin, but the levels of TLR4 were markedly increased after LY294002 treatment (*p* < 0.05, *F*[3,8] = 13.89) as shown by the immunohistochemistry staining of TLR4 in Fig. 4b second .

The western blot results showed that CUMS exposure robustly repressed the phosphorylation of PI3K (*p* < 0.005, *F*[3,12] = 23.11) and AKT (*p* < 0.01, *F*[3,12] = 28.53) and exacerbated the expression of TLR4 (*p* < 0.01, *F*[3,12] = 14.33) of the CUMS group mice versus the control group as described in Fig. 4c. The administration of baicalin (60 mg/kg) greatly elevated the expression of p-PI3K (*p* < 0.01) and p-AKT (*p* < 0.01) and significantly restrained the expression of TLR4 (*p* < 0.05) in CUMS mice. Nevertheless, LY294002 abolished the effects of baicalin by blocking the activation of PI3K/AKT and was responsible for the increase of TLR4 expression (*p* < 0.05) in the CUMS+LY+BA60 group. Collectively, these findings provide direct evidence that baicalin repressed the incremental expression of TLR4 and inflammatory responses induced by CUMS via activation of the PI3K/AKT pathway.

### Baicalin regulated CUMS-induced TLR4 expression by activating the PI3K/AKT/FoxO1 pathway in vivo

**Fig. 5:**
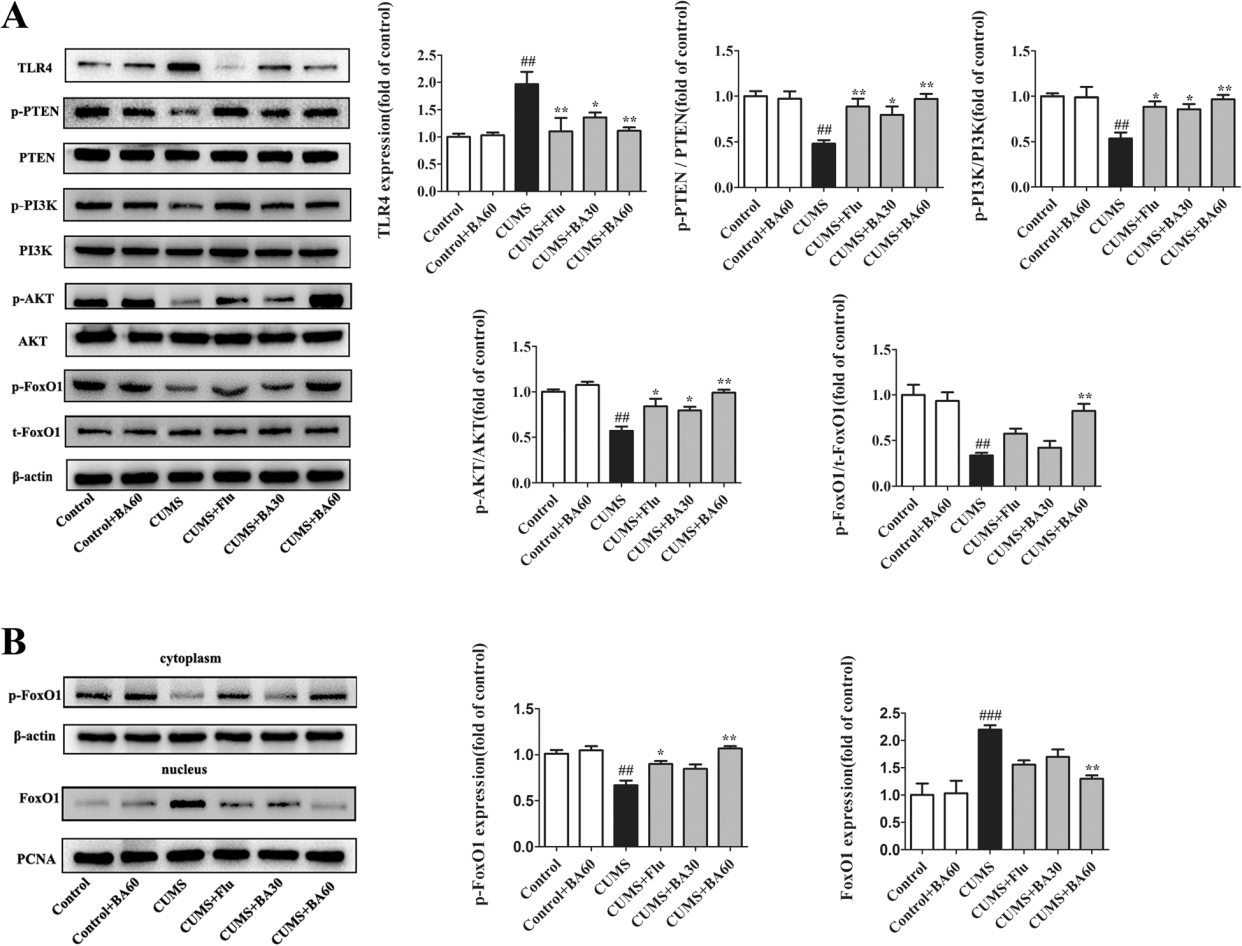
Effects of baicalin on the PI3K/AKT/FoxO1 pathway in the hippocampus of CUMS-induced mice. **a** Western blot was performed to detect the protein levels of TLR4, p-PTEN, p-PI3K, p-AKT, and p-FoxO1 in the hippocampus (*n* = 4). **b** The levels of p-FoxO1 in cytoplasmic fractions and FoxO1 in the nuclear extractions were determined by western blot (*n* = 4). All data were normalized to the control group and presented as means ± SEM. ^#^*p* < 0.05, ^##^*p* < 0.01, ^###^*p* < 0.005 vs. control; *p < 0.05, **p < 0.01 vs. CUMS

To further investigate the potential mechanism of baicalin on CUMS-evoked depression, the protein levels of PI3K/AKT-related signaling in the hippocampus of CUMS mice were examined by western blot. As shown in Fig. 5a and in comparison with the control group, CUMS robustly increased the expression of TLR4 (*p* < 0.01, *F*[5,18] = 6.188) and then substantially decreased the expression of p-PTEN (*p* < 0.01, *F*[5,18] = 7.265), p-PI3K (*p* < 0.01, *F*[5,18] = 6.778), p-AKT (*p* < 0.01, *F*[5,18] = 14.530), and p-FoxO1 (*p* < 0.01, *F*[5,18] = 16.240). Treatment with Flu (*p* < 0.05) and baicalin (60 mg/kg, *p* < 0.01) significantly reversed CUMS-elicited alterations in the expression of TLR4, p-PTEN, p-PI3K, and p-AKT compared with the CUMS group. As for p-FoxO1, although Flu did not completely alter the downtrend of p-FoxO1 expression, it still improved the situation induced by CUMS, and baicalin (60 mg/kg) absolutely changed the downtrend of p-FoxO1 expression induced by CUMS (*p* < 0.01). However, the Flu and baicalin (60 mg/kg) treatments had no significant effects on the levels of t-FoxO1 in CUMS mice.

FoxO1 is a transcriptional factor binding to multiple enhancer-like elements within the TLR4 gene [23], and its nuclear localization is modulated by phosphorylation. Thus, the subsequent experiments were designed to investigate the nuclear translocation of FoxO1 by detecting the levels of p-FoxO1 and FoxO1 in the cytoplasmic and nuclear fractions, respectively, of hippocampal tissues of CUMS mice. As exhibited in Fig. 5b, the levels of p-FoxO1 in the cytoplasmic protein extractions were strongly weakened after CUMS exposure in contrast to the control (*p* < 0.01, *F*[5,18] = 12.25), and treatment with Flu (*p* < 0.05) or baicalin (60 mg/kg, *p* < 0.01) increased the levels of p-FoxO1 in the cytoplasm compared with CUMS. Correspondingly, the levels of FoxO1 in the nuclear fractions extracted from the hippocampus of the CUMS group mice were significantly higher than those of the control group mice (*p* < 0.005, *F*[5,18] = 10.22). The CUMS-induced uptrend of the FoxO1 level in the nuclear extractions was dramatically reversed by the administration of baicalin (60 mg/kg, *p* < 0.01) and could be altered (to a certain extent) by the application of Flu. These data indicate that baicalin could indirectly promote the phosphorylation of FoxO1 and then promote its nuclear exclusion and subsequent sequestration into the cytoplasm in CUMS-induced mice, which was responsible for inhibiting FoxO1 transcription activity and then repressing the expression of TLR4.

**Fig. 6:**
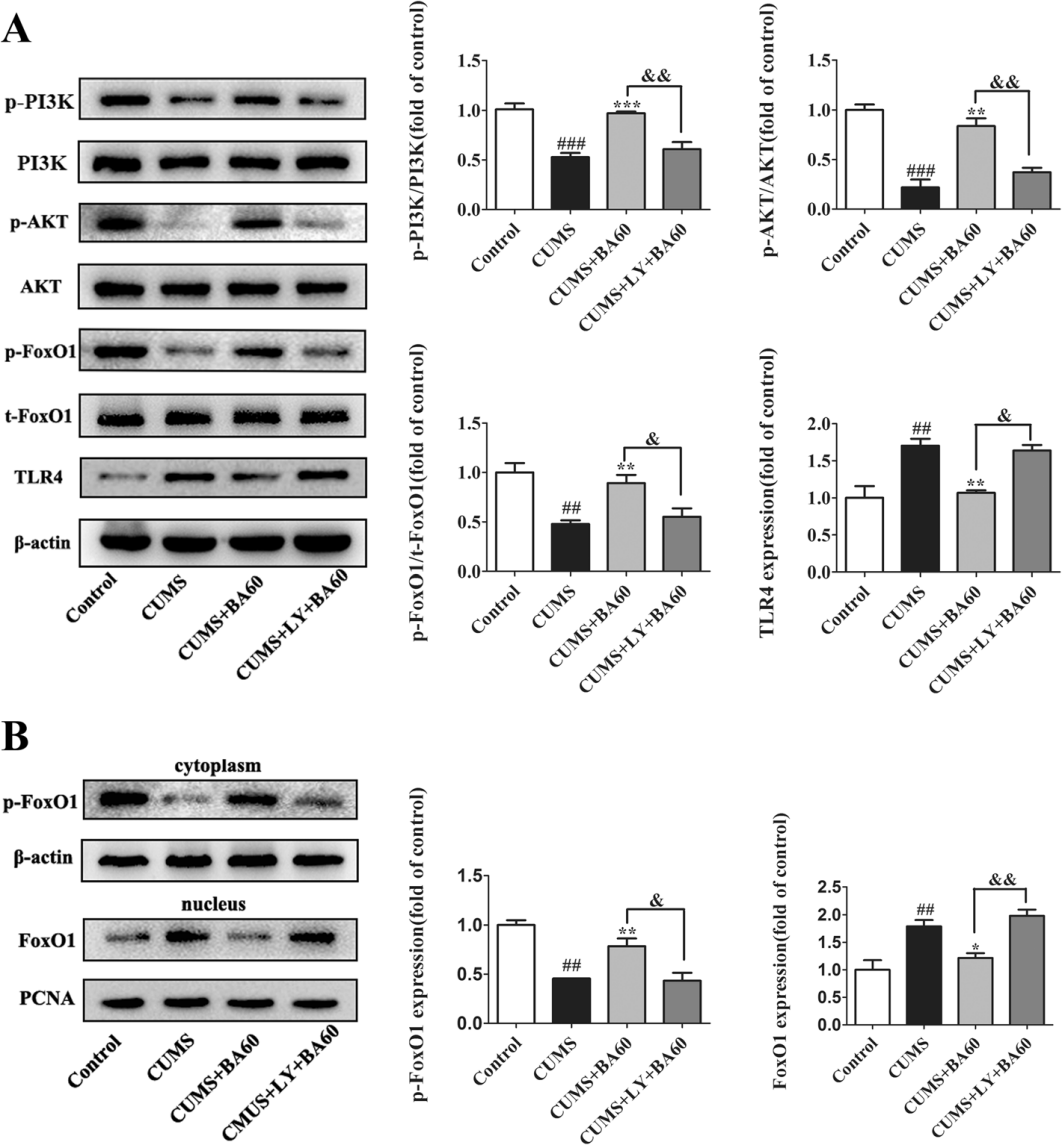
Baicalin inhibited FoxO1 transactivation and TLR4 expression in the hippocampus of CUMS mice in a manner dependent on PI3K/AKT. **a** Western blot was performed to determine the levels of p-PI3K, p-AKT, p-FoxO1, and TLR4 in the hippocampus of CUMS mice after treatment with baicalin and LY294002 (*n* = 4). **b** Representative protein bands of p-FoxO1 in the cytoplasmic fractions and FoxO1 in the nuclear extractions. Data were normalized to the control group and presented as means ± SEM. ^#^*p* < 0.05, ^##^*p* < 0.01, ^###^*p* < 0.005 vs. control; *p < 0.05, **p < 0.01 vs. CUMS; ^&^*p* < 0.05, ^&&^*p* < 0.01 vs. CUMS+BA60

To explore the role of PI3K/AKT signaling in the repression of TLR4 expression by baicalin, we employed LY294002 to block the PI3K/AKT pathway. From the western blot data in Fig. 6a, the inhibition of PI3K by LY294002 efficiently abrogated the effects of baicalin on p-PI3K (*p* < 0.01, *F*[3,12] = 22.16), p-AKT (*p* < 0.01, *F*[3,12]=39.36), p-FoxO1 (*p* < 0.05, *F*[3,12] = 10.50), and TLR4 (*p* < 0.05, *F*[3,12] = 17.43) expression in the hippocampus of the CUMS group mice. In addition, the inhibition of PI3K/AKT activation was responsible for the dephosphorylation of FoxO1 and increased its nuclear accumulation and activity as exhibited in Fig. 6b. Altogether, these results suggest that baicalin could inhibit FoxO1 transactivation via activation of PI3K/AKT signaling and then inhibit the expression of TLR4 in CUMS mice.

### Baicalin regulated LPS-induced TLR4 expression by activating the PI3K/AKT/FoxO1 pathway in BV2 cells

**Fig. 7:**
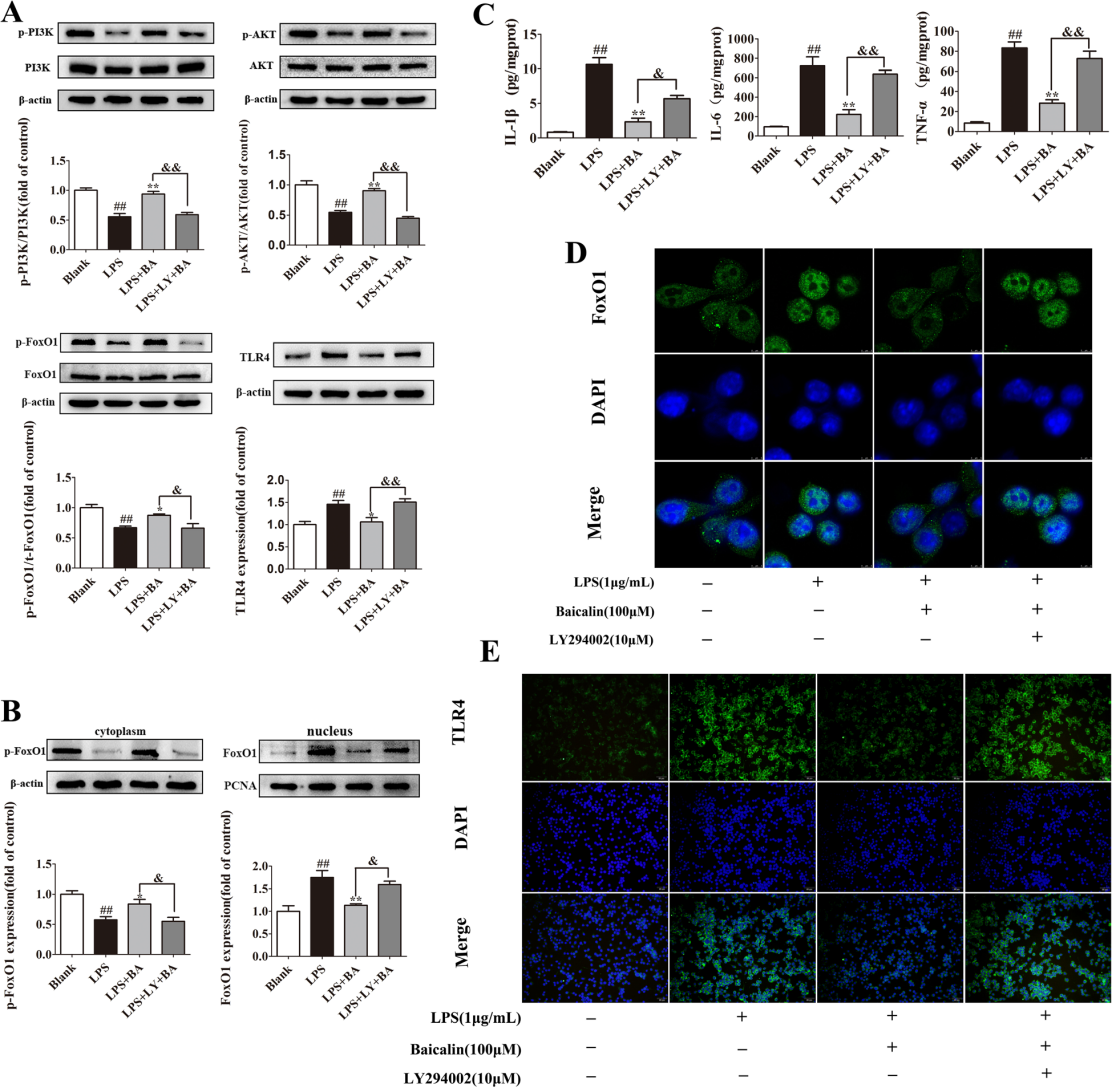
Baicalin regulates LPS-induced TLR4 expression by activating the PI3K/AKT/FoxO1 pathway in BV2 cells. **a**–**e** BV2 cells were pretreated with baicalin (100 μM) and LY292002 (10 μM) for 1 h and then stimulated with LPS (1 μg/mL) for 24 h. **a** Western blot was performed to measure the levels of p-PI3K, p-AKT, p-FoxO1, and TLR4. **b** The levels of p-FoxO1 in the cytoplasmic fractions and FoxO1 in the nuclear extractions (*n* = 4). **c** The levels of IL-1β, IL-6, and TNF-α in the cell culture medium were determined by ELISA kits (*n* = 8). Data were presented as means ± SEM. ^#^*p* < 0.05, ^##^*p* < 0.01 vs. blank; *p < 0.05, **p < 0.01 vs. LPS; ^&^*p* < 0.05, ^&&^*p* < 0.01 vs. LPS+BA. **d** The immunofluorescent images of the FoxO1 nucleus translocation were observed with confocal scanning microscopy in LPS-treated BV2 cells. Green, FoxO1; blue, DAPI; scale bar, 5 μm. **e** BV2 cells were stained with a TLR4 antibody and observed with fluorescence microscopy. Green, TLR4; blue: DAPI; scale bar, 50 μm

Microglial cells are the main source of pro-inflammatory factors in the brain after stress. Therefore, it is worthwhile to perform extracorporeal validation in BV2 microglial cells. The LPS, specifically combined with TLR4, was employed to trigger inflammatory responses in BV2 cells. As described in Fig. 7c, the BV2 cells were incubated with LPS (1 μg/mL) for 24 h, and the ensuing production of IL-1β (*p* < 0.01, *F*[3,28] = 51.86), IL-6 (*p* < 0.01, *F*[3,28] = 48.93), and TNF-α (*p* < 0.01, *F*[3,28] = 30.78) robustly increased in the LPS group versus the blank group. Pretreatment with baicalin (100 μM) could efficiently change their uptrend; however, LY294002 (10 μM) significantly abolished the effects of baicalin.

As the western blot results show in Fig. 7a, the expressions of p-PI3K (*p* < 0.01, *F*[3,12] = 26.46), p-AKT (*p* < 0.01, *F*[3,12] = 37.41), and p-FoxO1 (*p* < 0.01, *F*[3,12] = 11.18) were strongly weakened and the level of TLR4 (*p* < 0.01, *F*[3,12] = 10.09) was robustly higher after LPS administration in contrast to the blank group. The application of baicalin (*p* < 0.05) greatly reversed these alterations in the LPS-treated BV2 cells, and the protective effects of baicalin could be abolished by the incubation with LY294002.

Figure 7b demonstrates that LPS raised the activity of FoxO1 by increasing the dephosphorylation of FoxO1 and its nuclear accumulation. When the BV2 cells were treated with baicalin, the phosphorylation of FoxO1 was enhanced and its nuclear exclusion was promoted. However, the augmented effect of baicalin on FoxO1 phosphorylation was also weakened by LY294002. Likewise, Fig. 7d also showed that baicalin inhibited FoxO1 transcriptional activity in LPS-incubated BV2 cells by promoting FoxO1 nuclear exclusion and then substantially decreased the LPS-induced TLR4 expression (Fig. 7e). The application of LY294002 abolished the effect of baicalin on FoxO1 nuclear exclusion (Fig. 7d) and then substantially increased the expression of TLR4 (Fig. 7e). Collectively, these results demonstrate that baicalin downregulated the LPS-induced TLR4 expression by activating the PI3K/AKT/FoxO1 pathway in BV2 cells.

### Baicalin regulated LPS-induced TLR4 expression by activating the PI3K/AKT/FoxO1 pathway in primary microglia cells

**Fig. 8:**
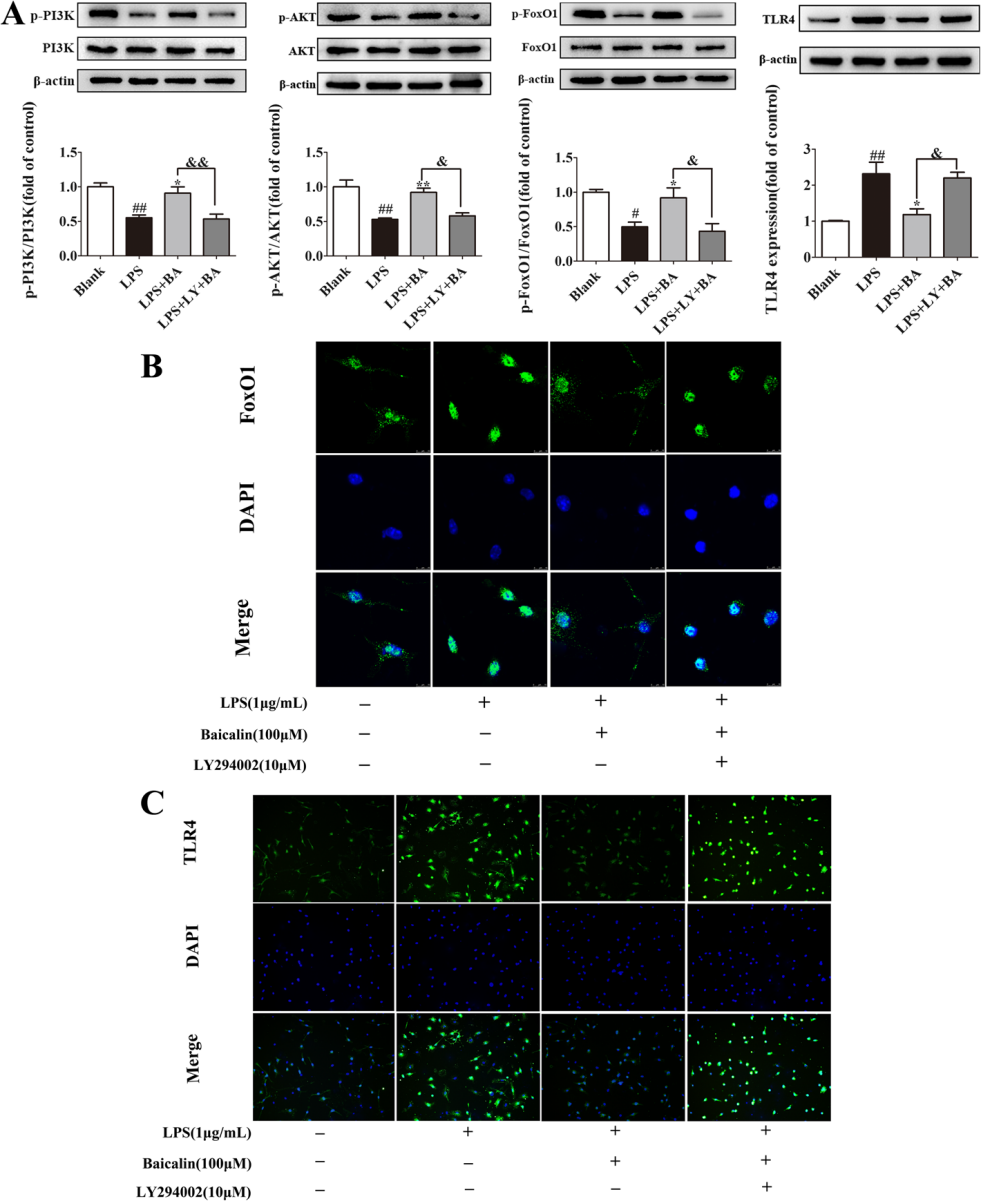
Baicalin regulates LPS-induced TLR4 expression by activating the PI3K/AKT/FoxO1 pathway in primary microglia cells. Primary microglia cells were pretreated with baicalin and LY 294002 for 1 h and then stimulated with LPS for 24 h. **a** Western blot was performed to measure the levels of p-PI3K, p-AKT, p-FoxO1, and TLR4. Data were normalized to the blank group and presented as means ± SEM. ^#^*p* < 0.05, ^##^*p* < 0.01 vs. blank; *p < 0.05, **p < 0.01 vs. LPS; ^&^*p* < 0.05, ^&&^*p* < 0.01 vs. LPS+BA. **b** The immunofluorescent images of FoxO1 nucleus translocation were observed by confocal scanning microscopy in LPS-treated primary microglia cells. Green, FoxO1; blue: DAPI; scale bar 10 μm. **c** Primary microglia cells were stained with a TLR4 antibody and observed with fluorescence microscopy. Green, TLR4; blue, DAPI; scale bar 50 μm

To confirm the data obtained from BV2 microglial cells, we further proved the above findings in primary mouse microglia cells. As the western blot results described in Fig. 8a, in response to LPS stimulation, we observed a decrease in the protein levels of p-PI3K (*p* < 0.01, *F*[3,8] = 12.81), p-AKT (*p* < 0.01, *F*[3,8] = 14.25), and p-FoxO1 (*p* < 0.05, *F*[3,8] = 8.319) and an increase of TLR4 (*p* < 0.01, *F*[3,8] = 11.67) in LPS-treated primary microglia cells. Pretreatment with baicalin (100 μM) resulted in a significant upregulation of p-PI3K (*p* < 0.05), p-AKT (*p* < 0.01), and p-FoxO1 (*p* < 0.05). Since the phosphorylation of FoxO1 was enhanced, the activity of FoxO1 was inhibited, which resulted in its nuclear exclusion in primary microglia cells as shown in Fig. 8b. In addition, the regulating transcriptional effects of FoxO1 on TLR4 (*p* < 0.05) gene expression were also inhibited by baicalin as exhibited in Fig. 8a and c. However, application of LY294002 (10 μM) abolished the effect of baicalin on FoxO1 nuclear exclusion (Fig. 8b) and on downregulation expression of TLR4 (Fig. 8a, c). These results reconfirmed that the anti-neuroinflammatory effect of baicalin was mediated by the PI3K/AKT/FoxO1 pathway. Surprisingly, these results were mainly consistent with the experiments described above and again indicate that baicalin regulated the incremental expression of TLR4 and inflammatory responses induced by stress via activation of the PI3K/AKT/FoxO1 pathway.

### FoxO1 played a vital role in baicalin regulation of the TLR4 signaling

**Fig. 9:**
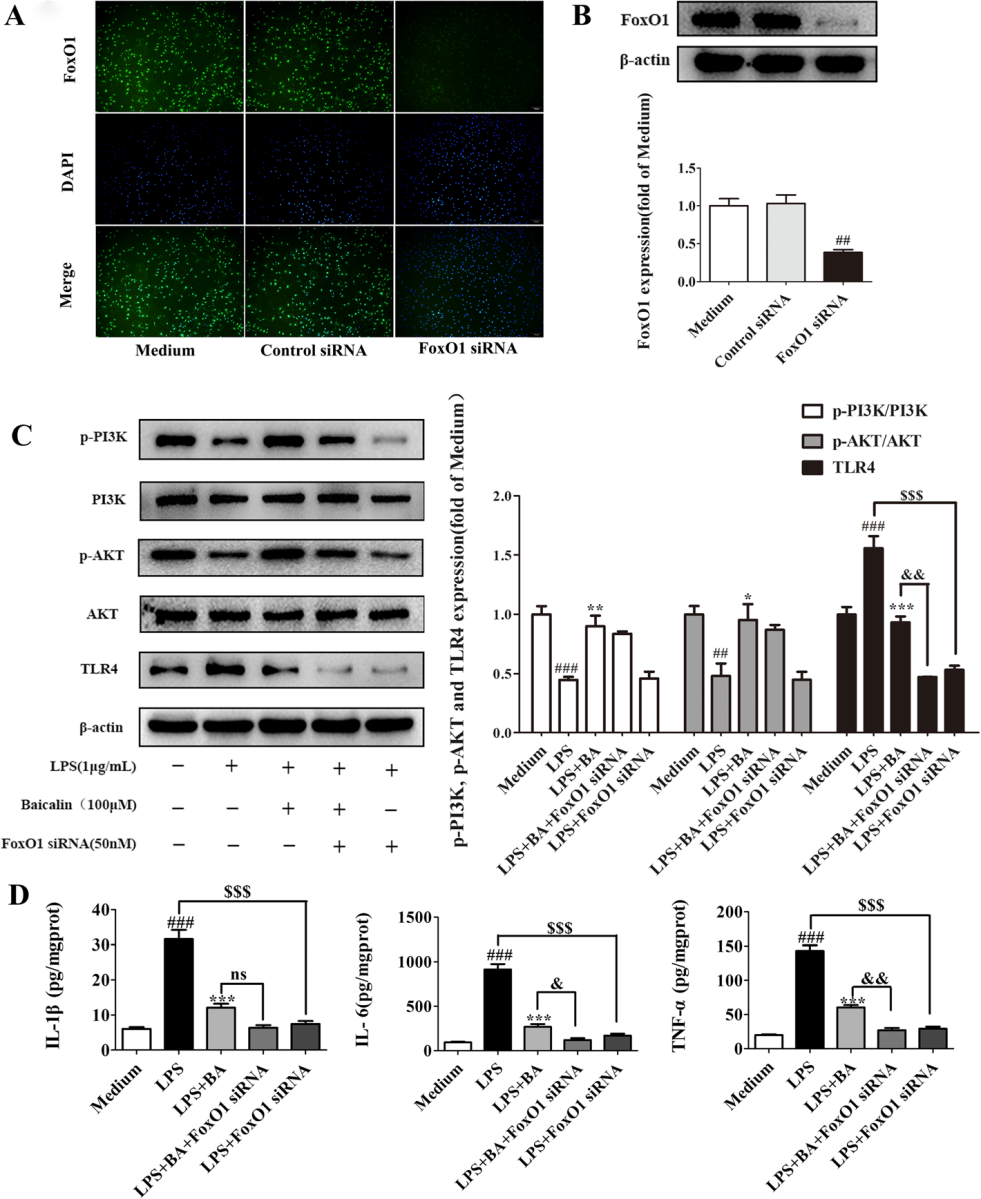
The knockdown of FoxO1 enhanced the inhibitory effect of baicalin on LPS-induced TLR4 expression and inflammation. **a**–**d** The knockdown of FoxO1 expression by FoxO1 siRNA. **a** The cells were stained with FoxO1 (green) antibody and observed using fluorescence microscopy (scale bar 100 μm). **b** Western blotting analysis of the expression of FoxO1 after the suppression of FoxO1 expression. **c** Western blotting analysis of the expression of p-PI3K, p-AKT, and TLR4 in LPS-stimulated primary microglia after transfection with FoxO1 siRNA and treated or not treated with baicalin. The levels of p-PI3K, p-AKT, and TLR4 were quantified. Data were normalized to the medium group. **d** The levels of IL-1β, IL-6, and TNF-α in the cell culture supernatants of LPS-stimulated primary microglia were determined by ELISA after transfection with FoxO1 siRNA. All of the results are presented as means ± SEM. ^#^*p* < 0.05, ^##^*p* < 0.01 vs. medium; *p < 0.05, **p < 0.01 vs. LPS; ^&^*p* < 0.05, ^&&^*p* < 0.01 vs. LPS+BA60; ^$$$^*p* < 0.005 vs. LPS

**Fig. 10:**
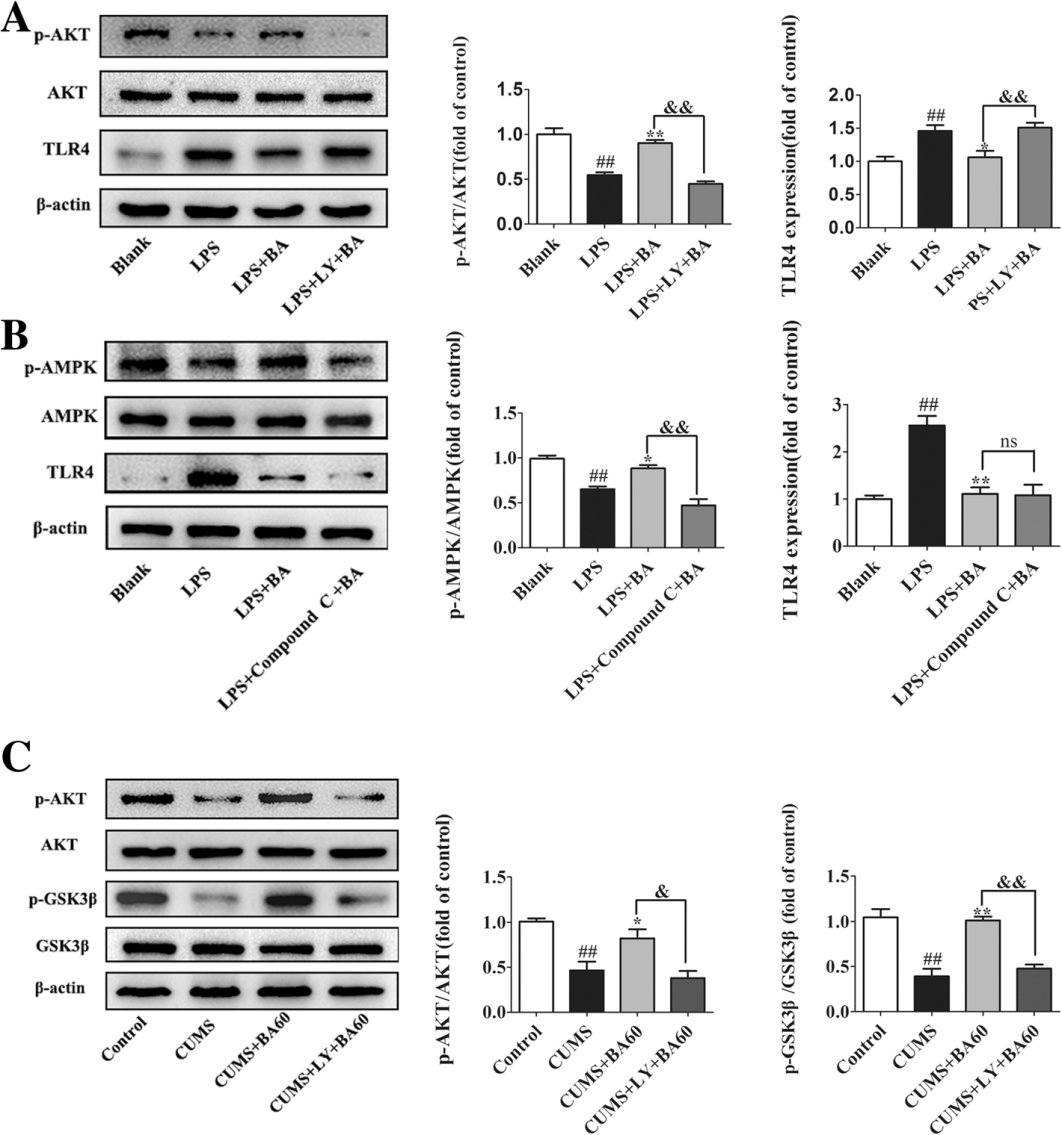
A discussion of the potential targets of baicalin. **a**, **b** BV2 cells were pretreated with LY 292002 (10 μM) and compound C (20 μM) and co-incubated with baicalin (100 μM) for 1 h and then stimulated with LPS (1 μg/ml) for 24 h. Western blot was performed to detect the protein levels of p-AKT, TLR4 (**a**, *n* = 4), p-AMPK, and TLR4 (**b**, *n* = 4), and a statistical analysis was conducted. Data were normalized to the blank group and presented as means ± SEM. *p < 0.05, **p < 0.01 vs. blank; ^#^*p* < 0.05, ^##^*p* < 0.01 vs. LPS; ^&^*p* < 0.05, ^&&^*p* < 0.01 vs. LPS+BA60. **c** Western blot was performed to measure the levels of p-AKT and p-GSK3β in the hippocampus of CUMS mice after treatment with LY294002 and baicalin (*n* = 4). Data are presented as means ± SEM. *p < 0.05, **p < 0.01 vs. control; ^#^*p* < 0.05, ^##^*p* < 0.01 vs. CUMS; ^&^*p* < 0.05, ^&&^*p* < 0.01 vs. CUMS+BA60

The transcriptional activity of FoxO1 was inhibited via phosphorylation by PI3K/AKT, which leads to the nuclear translocation and inhibition of target gene expression. To verify whether FoxO1 was involved in baicalin regulation of the TLR4 signaling, we knocked down the expression of FoxO1 using RNA interference (RNAi) based on the primary microglia cells. The primary microglia cells were transfected with FoxO1-siRNA for 5 h, and after resting overnight, the cells were treated with baicalin and LPS for 24 h. The FoxO1 was successfully inhibited by FoxO1-siRNA compared with the control-siRNA (*p* < 0.01, *F*[2, 9] = 17.31) as well as non-treated cells (*p* < 0.01) (Fig. 9a, b). As described in Fig. 9c, the transfection of FoxO1-siRNA significantly strengthened the effects of baicalin on LPS-induced TLR4 expression (^&&^*p* < 0.01, *F*[4,15] = 59.42), while it had no significant effects on the expression of p-PI3K and p-AKT. These results indicate that FoxO1 is a crucial downstream target of AKT. However, the increases of the TLR4 levels induced by LPS were abrogated by FoxO1-siRNA (^$$$^*p* < 0.005). Likewise, the production of IL-1β (^$$$^*p* < 0.005, *F*[4,25] = 59.23), IL-6 (^$$$^*p* < 0.005, *F*[4,25] = 104.9), and TNF-α (^$$$^*p* < 0.005, *F*[4,25] = 133.0) induced by LPS in primary microglia cells was also abolished by FoxO1-siRNA as shown in Fig. 10d. Meanwhile, the FoxO1-siRNA significantly strengthened the inhibitory effects of baicalin on the LPS-induced production of IL-6 (^&^*p* < 0.05) and TNF-α (^&&^*p* < 0.01) in primary microglia cells. Thus, it could be inferred that FoxO1 played a vital role in the inhibitory effect of baicalin on TLR4 expression and TLR4-mediating inflammation signaling triggered by LPS.

## Discussion

The anti-inflammatory effects of baicalin in peripheral inflammatory diseases have been broadly investigated and might be linked to the inhibition of TLR4 and its downstream signaling [25–28]. Current research studies indicate that neuroinflammation plays a vital role in the pathogenesis of depressive disorders [9, 57]. In this study, we found that the chronic application of baicalin exhibited antidepressant effects by ameliorating neuroinflammation in the CUMS model of depressive mice, and the underlying mechanisms were associated with the inhibition of TLR4 expression via the PI3K/AKT/FoxO1 pathway.

Considering that stress induced the production of pro-inflammatory cytokines, which might evoke a depressive-like state in animal model systems [3], it is reasonable to presume that stress-induced increases of inflammation might play a role in depression. In our study, the levels of IL-1β, IL-6, and TNF-α were substantially increased in the hippocampus of the depression mice model induced by CUMS. However, the administration of baicalin (60 mg/kg) apparently ameliorated CUMS-induced depressive-like symptoms and greatly decreased pro-inflammatory cytokines in the hippocampus while concurrently downregulating TLR4 expression. These data allude to the fact that baicalin could inhibit neuroinflammation and TLR4 might participate in this process.

TLR4 regulates the adrenal responses to stress and inflammatory stimuli as well as brain responses to stress [15, 16]. Previous studies demonstrated that stress exposure upregulated the TLR4 pathway, and this suggests a vital role of TLR4 in the production of neuroinflammation in the brain [58]. In this study, we substantiated the fact that TLR4 indeed participated in the anti-neuroinflammatory and antidepressive effects of baicalin as follows: (i) activation of TLR4 by LPS-triggered robust elevation of IL-1β, IL-6, and TNF-α in the hippocampus and injury of hippocampal neurons as well as depressive symptoms in mice; (ii) blocking TLR4 by TAK-242 reversed LPS-induced neuroinflammation and depressive-like behaviors; and (iii) baicalin efficiently inhibited neuroinflammation and the expression of TLR4 induced by the specific activation of TLR4 with LPS, and baicalin also ameliorated behavioral changes induced by LPS with a similar efficacy of TAK-242.

Previous studies reported that the activation of TLR4 by LPS or HMGB1 resulted in the activation of PTEN through dephosphorylation [49, 59, 60]. PTEN is a tumor suppressor with dual phosphatase activity and expression in the neurons in the human, mouse, and rat brain, and has a functional role in the CNS development by regulating cell senescence, apoptosis, inflammation, and other pathophysiological processes [61]. PTEN is known to be a major negative regulator of PI3K/AKT by dephosphorylating PIP3 to PIP2, which results in antagonizing PI3K and then inactivating AKT [62, 63]. Accordingly, our results demonstrate that the CUMS stressors activated TLR4, which led to the increase of TLR4 and the decrease of p-PTEN (inactive form) expression and promoted the activation of PTEN and then inhibition of the phosphorylation of AKT (Fig. 5). However, treatment with baicalin decreased the expression of TLR4 and elevated the expression of p-PI3K and p-AKT, which could be related to the activation of the PI3K/AKT signaling pathway. Interestingly, previous studies indicated that the phosphorylation of PI3K/AKT, as a downstream target of TLR4, was dependent on LPS-stimulated TLR4 activation in a time-dependent manner and reached a maximum level 1 h after LPS stimulation [55, 64]. This might be a transient self-limiting feedback mechanism. However, in CUMS mice, accumulating PTEN might play a predominant role in the phosphorylation of PI3K and AKT and inhibit PI3K and AKT phosphorylation. Then, the total level of p-PI3K and p-AKT is decreased.

FoxO1, which is a member of the forkhead transcription factor forkhead box protein O (FoxO) family, has important functions in cellular processes including DNA repair, cell cycle control, stress resistance, apoptosis, and cell metabolism [65, 66]. FoxO1 is also a transcriptional regulator of TLR4 and its pro-inflammatory pathway by binding to multiple enhancer-like elements within the TLR4 gene itself as well as to sites in several TLR4 signaling pathways [23]. Previous research has demonstrated that FoxO1 promotes the expression of IL-6, TNF-α, and IL-1β in LPS-induced macrophages by directly transactivating the TLR4 gene and then upregulating the TLR4 gene expression [23]. Moreover, the activity of FoxO1 was mediated via phosphorylation by PI3K/AKT. The phosphorylation of FoxO1 by PI3K/AKT was able to reduce its transactivation potential, which leads to the blockage of DNA binding, nuclear exclusion, and subsequent sequestration into the cytoplasm and thus results in the inhibition of target gene expression [24, 67]. Consistent with previous findings, we demonstrated that the baicalin treatment greatly increased the expression of p-PI3K and p-AKT and then promoted the phosphorylation of FoxO1, contributed to FoxO1 nuclear exclusion, and finally resulted in attenuating the expression of TLR4 in the CUMS group mice as shown in Fig. 5 and Fig. 6. However, LY294002, which is a PI3K inhibitor, abolished the above effects of baicalin by inhibiting the activation of PI3K/AKT. Therefore, we depicted the molecular mechanisms in which baicalin might regulate the expression of TLR4 in CUMS mice via activation of the PI3K/AKT/FoxO1 pathway. Meanwhile, the effect of baicalin mediating the expression of TLR4 via the PI3K/AKT/FoxO1 pathway was also observed in LPS-induced inflammatory injury in BV2 cells and primary microglia cells (Fig. 7 and Fig. 8). Importantly, FoxO1, which is a transcriptional regulator, regulated TLR4 and its pro-inflammatory pathway in LPS-treated microglia cells and played a vital role in the inhibitory effect of baicalin on the LPS-simulated expression of TLR4 and TLR4-mediating neuroinflammation (Fig. 9).

Baicalin is a lipophilic flavonoid glycoside that can pass through the blood-brain barrier (BBB) and has therapeutic effects on the central nervous system [19, 68, 69] including ameliorating depression [70] and cerebral ischemia [18, 20]. We found that the anti-neuroinflammatory effect of baicalin in depressed mice was associated with downregulating TLR4 expression via activation of the PI3K/AKT pathway. However, Li et al. found that baicalin attenuated ischemia/reperfusion injury by regulating mitochondrial function in a manner dependent on AMPK [20]. AMPK involvement in downregulating TLR4 expression by baicalin had not been previously elucidated. To further this investigation, we employed LY294002 and compound C to specifically inhibit PI3K and AMPK in baicalin-treated BV2 cells, respectively. As Fig. 10a and b shows, the effect of baicalin on LPS-stimulated TLR4 expression could be abolished by LY294002 rather than compound C. Hence, baicalin might attenuate ischemia/reperfusion injury depending on AMPK, but downregulated the expression of TLR4 via PI3K/AKT instead of AMPK. Recently, Zhang et al. discovered that baicalin exerts neuroprotective effects via inhibiting the activation of GSK3β in a rat model of depression [71]. There is evidence suggesting that glycogen synthase kinase-3 (GSK3) β is a downstream molecule of PI3K/AKT [72], and the activation of PI3K/AKT results in the increase of p-GSK3β and then inhibits the activation of GSK3β. We also investigated this molecular mechanism in CUMS mice as described in Fig. 10c. CUMS exposure repressed the phosphorylation of AKT (*p* < 0.01, *F*[3,12] = 12.84) and GSK3β (*p* < 0.01, *F*[3,12] = 24.34) in mice, and the administration of baicalin greatly elevated the expression of p-AKT and p-GSK3β. LY294002 not only inhibits the phosphorylation of AKT, but also inhibits the phosphorylation of GSK3β, which was consistent with a previous study showing that baicalin could inhibit the activation of GSK3β. Interestingly, we also determined that baicalin inhibits the activation of GSK3β via the PI3K/AKT pathway.

## Conclusions

**Fig. 11:**
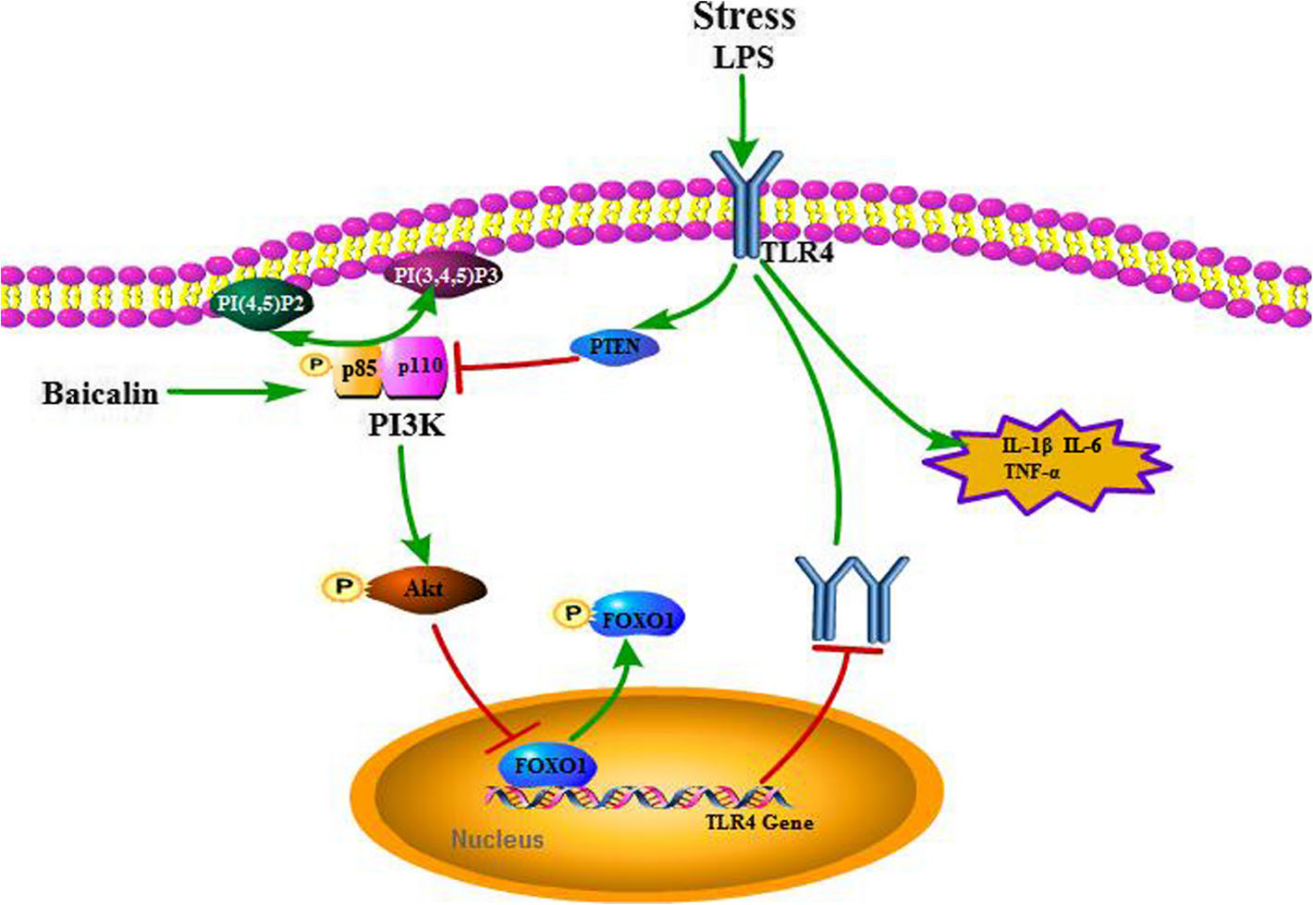
A schematic illustration of the proposed mechanism for baicalin to restrain neuroinflammation

The proposed pathway is shown in Fig. 11, and we demonstrated that baicalin could improve depressive-like behavior and ameliorate neuroinflammation in a CUMS and LPS-induced mice model of depression via the inhibition of pernicious overexpression of TLR4 via the PI3K/AKT/FoxO1 pathway. In addition, our results elucidated the underlying mechanisms of baicalin on the regulation of TLR4 expression in CUMS mice for the first time.

## Additional file

Additional file 1: The details of primary mouse microglial cell culture. **Figure S1.** Iba-1 immunostaining images and morphology pictures of primary microglias isolated using mild trypsinization. Scale bar = 50 μm (TIF 7.29 mb). **Figure S2.** Iba-1 immunostaining images and morphology pictures of primary microglia isolated using shaking. The scale bar = 50 μm and arrows refer to the enlarged round cell body. (ZIP 24043 kb)

## Abbreviations

AKT: Protein kinase B
AMPK: 5′ adenosine monophosphate-activated protein kinase
CSF: Cerebrospinal fluid
CUMS: Chronic unpredictable mild stress
DMEM: Dulbecco’s modified Eagle’s medium
DMEM/F12: Dulbecco’s modified Eagle’s medium nutrient mixture F-12
ELISA: Enzyme-linked immunosorbent assay
FBS: Fetal bovine serum
Flu: Fluoxetine
FoxO1: Forkhead box O1
FST: Forced swimming test
GSK3β: Glycogen synthase kinase-3 (GSK3) β
Iba1: Ionized calcium-binding adapter molecule 1
IL-1β: Interleukin-1 beta
IL-6: Interleukin-6
LPS: Lipopolysaccharide
MDD: Major depressive disorder
OFT: Open field test
PBS: Phosphate buffer saline
PI3K: Phosphatidylinositol 3-kinase
PIP2: Phosphatidylinositol 3,4-bisphosphate (PI (3,4)P2)
PIP3: Phosphatidylinositol (3,4,5)-trisphosphate (PI(3,4,5)P3)
SPT: Sucrose preference test
TLR4: Toll-like receptor 4
TNF-α: Tumor necrosis factor alpha
TST: Tail suspension test
