# Supplementary material for: Baicalin ameliorates neuroinflammation-induced depressive-like behavior through inhibition of toll-like receptor 4 expression via the PI3K/AKT/FoxO1 pathway
Source: J Neuroinflammation. 2019 May 8;16:95. doi: 10.1186/s12974-019-1474-8 (PMC6507025; doi:10.1186/s12974-019-1474-8)
Supplement: Supplementary file 1 — The details of primary mouse microglial cell culture. Figure S1. Iba-1 immunostaining images and morphology pictures of primary microglias isolated using mild trypsinization. Scale bar = 50 μm (TIF 7.29 mb). Figure S2. Iba-1 immunostaining images and morphology pictures of primary microglia isolated using shaking. The scale bar = 50 μm and arrows refer to the enlarged round cell body. (ZIP 24043 kb) [file 12974_2019_1474_MOESM1_ESM.zip › Supplemental Material.docx]

**Supplemental Material**

**Primary mouse microglial cells culture**

Primary microglia cultures were prepared from hippocampal tissues of 1-day-old neonatal ICR mice as described early with some modifications [1,2,3,4]. Briefly, hippocampal tissues, devoid of meninges and blood vessels, were digested with 0.125% Trypsin-EDTA for 15 min at 37°C, followed by mechanical triturating in Dulbecco’s modified eagle’s medium nutrient mixture F-12 (DMEM/F12, Gibco) with 10% fetal bovine serum (FBS,Gibco). After centrifugation (1000 rpm, 10 min), cells were collected and re-suspended in DMEM/F12 with 10% FBS. Then the mixed cells were passed through a 70 μm mesh cell strainer and plated in DMEM/F12 containing 10% FBS and antibiotics (40 U/mL penicillin and 40 μg/mL streptomycin). The mediums were completely replaced every 3-4 days and confluency was achieved after about 10-12 days in vitro (DIV).

There are two commonly used methods to purify microglia involved shaking and mild trypsiniztion [5,6]. We used mild trypsinization method to isolated microglia from mixed glial cultures after comparing microglia obtained with shaking versus trypsinization. Briefly, after 15-18 DIV, incubation of mixed glial cultures with a trypsin solution (0.25% trypsin-EDTA diluted 1:4 in DMEM/F12) for 30 – 45 min at 37 °C resulted in the detachment of an intact layer of cells in one piece, whereas microglial cells remained attached to the bottom of the well. The specificity and purity of the cultured microglial cell has been confirmed by immunostaining with Iba1 (Additional file 2: Figure S1). We also purified microglia by shaking [7], but the morphology of microglia was more heterogeneous than mild trypsinization method, and most of them showed enlarged round cell body. (Additional file 3: Figure S2).

Figure S1. Iba-1 immunostaining images and morphology pictures of primary microglias isolated using mild trypsinization. Scale bar = 50 μm

Figure S2. Iba-1 immunostaining images and morphology pictures of primary microglia isolated using shaking. Scale bar = 50 μm. Arrows refer to enlarged round cell body

**References:**

1. Liu D, Wang Z, Liu S, Wang F, Zhao S, Hao A. Anti-in fl ammatory effects of fluoxetine in lipopolysaccharide ( LPS ) -stimulated microglial cells. Neuropharmacology. 2011;61:592–599. doi.org/10.1016/j.neuropharm.2011.04.033.
2. Gao HM, Hong JS, Zhang W, Liu B. Distinct role for microglia in rotenone - induced degeneration of dopaminergic neurons. J Neurosci.2002;22:782–90. doi.org/10.1523/JNEUROSCI.22-03-00782.2002.
3. Saliba SW, Marcotegui AR, Fortwängler E, Ditrich J, Perazzo JC, Muñoz E, et al. AM404, paracetamol metabolite, prevents prostaglandin synthesis in activated microglia by inhibiting COX activity. J Neuroinflammation. 2017;14:DOI: 10.1186/s12974-017-1014-3.
4. Zhong L, Jiang X, Zhu Z, Qin H, Dinkins MB, Kong JN, et al. Lipid transporter Spns2 promotes microglia pro-inflammatory activation in response to amyloid-beta peptide. Glia. 2018; doi: 10.1002/glia.23558.
5. Saura J, Tusell JM, Serratosa J. High-Yield Isolation of Murine Microglia by Mild Trypsinization. Glia. 2003;44:183–189. doi.org/10.1002/glia.10274.
6. Lin L, Desai R, Wang X, Lo EH, Xing C. Characteristics of primary rat microglia isolated from mixed cultures using two different methods. Journal of Neuroinflammation. 2017;14: doi: 10.1186/s12974-017-0877-7.
7. Dong H, Zhang W, Zeng X, Hu G, Zhang H, He S, Zhang S. Histamine induces upregulated expression of histamine receptors and increases release of inflammatory mediators from microglia. Mol Neurobiol. 2014;49:1487–1500. doi.org/10.1007/s12035-014-8697-6.
