# Supplementary figures and images for: Baicalin ameliorates neuroinflammation-induced depressive-like behavior through inhibition of toll-like receptor 4 expression via the PI3K/AKT/FoxO1 pathway
Source: J Neuroinflammation. 2019 May 8;16:95. doi: 10.1186/s12974-019-1474-8 (PMC6507025; doi:10.1186/s12974-019-1474-8)

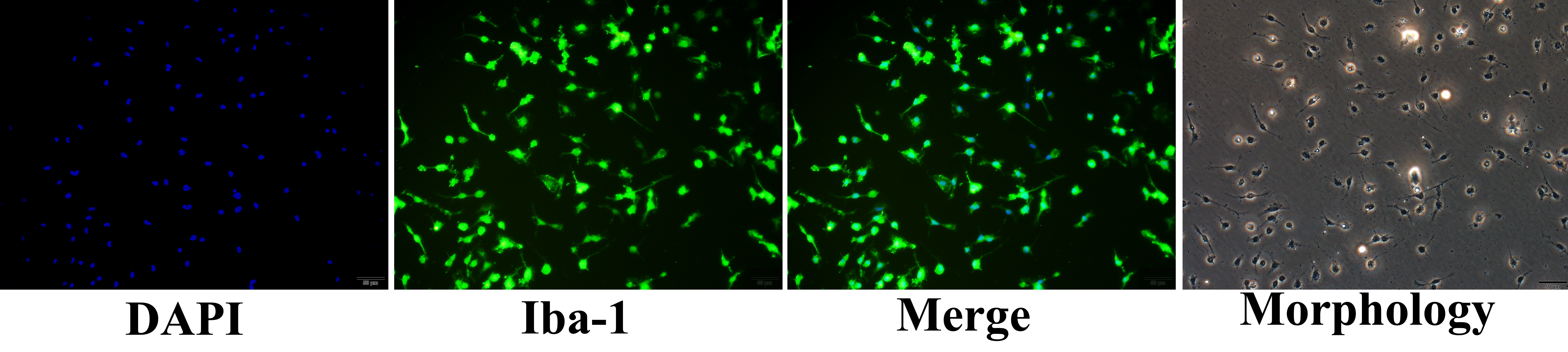

Supplement: Supplementary file 1 — The details of primary mouse microglial cell culture. Figure S1. Iba-1 immunostaining images and morphology pictures of primary microglias isolated using mild trypsinization. Scale bar = 50 μm (TIF 7.29 mb). Figure S2. Iba-1 immunostaining images and morphology pictures of primary microglia isolated using shaking. The scale bar = 50 μm and arrows refer to the enlarged round cell body. (ZIP 24043 kb) [file 12974_2019_1474_MOESM1_ESM.zip › Figure S1.tif]

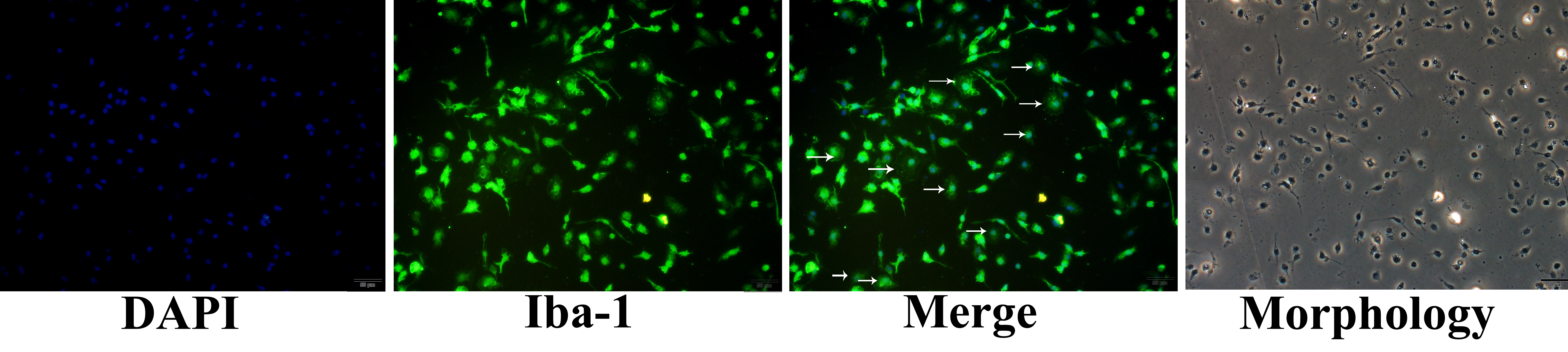

Supplement: Supplementary file 1 — The details of primary mouse microglial cell culture. Figure S1. Iba-1 immunostaining images and morphology pictures of primary microglias isolated using mild trypsinization. Scale bar = 50 μm (TIF 7.29 mb). Figure S2. Iba-1 immunostaining images and morphology pictures of primary microglia isolated using shaking. The scale bar = 50 μm and arrows refer to the enlarged round cell body. (ZIP 24043 kb) [file 12974_2019_1474_MOESM1_ESM.zip › Figure S2.tif]
